# Faecal bacterial and short-chain fatty acids signature in hypercholesterolemia

**DOI:** 10.1038/s41598-019-38874-3

**Published:** 2019-02-11

**Authors:** A. B. Granado-Serrano, M. Martín-Garí, V. Sánchez, M. Riart Solans, R. Berdún, I. A. Ludwig, L. Rubió, E. Vilaprinyó, M. Portero-Otín, J. C. E. Serrano

**Affiliations:** 1 Department of Experimental Medicine, University of Lleida, Lleida, Spain; 2 Institut Català de la Salut, Catalunya, Spain; 3 Functional Nutrition, Oxidation and Cardiovascular Disease Group, Faculty of Medicine and Health Sciences, Rovira i Virgili University, Reus, Spain; 4 Department of Food Technology, XaRTA-TV, Agrotecnio Center, High Technical School of Agricultural Engineering, University of Lleida, Lleida, Spain; 5 Department of Basic Medical Sciences, University of Lleida, Lleida, Spain

## Abstract

Gut microbiota has been suggested to affect lipid metabolism. The objective of this study was to characterize the faecal microbiota signature and both short chain fatty acids (SCFAs) and bile acids (BA) profile of hypercholesterolemic subjects. Microbiota composition, SCFAs, BA and blood lipid profile from male volunteers with hypercholesterolemia (HC) and normocholesterolemia (NC) were determined by 16S rDNA sequencing, HPLC, GC and NMR, respectively. HC subjects were characterized by having lower relative abundance of *Anaeroplasma* (0.002% vs 0.219%, p-value = 0.026) and *Haemophilus* (0.041% vs 0.078%, p-value = 0.049), and higher of *Odoribacter* (0.51% vs 0.16%; p-value = 0.044). Correlation analysis revealed that *Anaeroplasma* and *Haemophilus* were associated to an unfavourable lipid profile: they correlated negatively to cholesterol and triglycerides related biomarkers and the ratio total to high density lipoprotein (HDL) cholesterol, and positively to HDL size. *Odoribacter* displayed an opposite behaviour. Faecal SCFAs profile revealed higher abundance of isobutyric (2.76% vs 0.82%, p-value = 0.049) and isovaleric acid (1.32% vs 0.06%, p-value = 0.016) in HC. Isobutyric acid correlated positively with *Odoribacter* and lipid parameters indicative of an unfavourable profile. BA profile did not show differences between groups. It was concluded that HC subjects showed a particular faecal bacterial signature and SCFAs profile associated with their lipid profile.

## Introduction

Cardiovascular diseases (CVDs) remain the biggest cause of deaths globally. More than 17.7 million people died of CVDs in 2015, representing 31% of all death worldwide^1^. Hypercholesterolemia is considered an important modifiable risk factor of CVDs. Just in Spain, the prevalence of high total cholesterol (Total-C) levels (over 200 mg/dL) is 50–60%^2^, which makes it one of the most prevalent risk factor only surpassed in some cases by arterial hypertension^1^. Although some cases of hypercholesterolemia are due to genetic alterations (mutations of APOB or low density lipoprotein (LDL) receptor genes)^3^, most of them are related to lifestyle factors, such as diet and exercise, and other related metabolic pathologies, such as overweight, obesity or diabetes^4,5^. Therefore, most therapeutic strategies currently used against hypercholesterolemia are a combination of lipid-lowering medications and lifestyle modifications, especially dietary restrictions of fat and carbohydrates, addressed to reduce plasmatic lipids levels, whose main target is the levels of cholesterol carried by LDL (LDL-C)^6–8^. Other strategies are addressed to increase the levels of cholesterol carried by high density lipoprotein (HDL-C), which has been linked to a decrease in cardiovascular (CV) events. However, all these strategies have lower efficacy than expected, probably due to wrong selection of the target or inter-individual responses related to physiology.

Regarding the former, in last years, some dynamic studies defend that the particle size (Z) of lipoprotein may be better predictive factor of CV events and therefore, they could be a better target for strategies against hypercholesterolemia. Thus, a higher amount of large-HDL particles (large-HDL-P) and a larger average size of HDL (HDL-Z) have been positively related to a decrease of CV events, and the amount of small HDL particles (small-HDL-P) with an increase^9–12^. Whereas, with respect to the latter, gut microbiota could be relevant, since its study in last decade have proposed it as a novel key player in this pathology. The human gut harbours about 100 trillion of typically non-pathogenic microbes whose whole microbiome is closed to the staggering number of 5 million genes, which confers it a big capability to modify and regulate the host physiology. Increasing evidences, mainly from studies with germ free mice, support an important role of gut microbiota in host energy metabolism and blood lipid levels modulation^13,14^. These microbes and their metabolic products such as short chain fatty acids (SCFAs), or bile acids (BA), etc. have been reported to influence the efficiency of energy harvesting, as well as activate the immune system, modulate the chronic inflammation through the alteration of intestinal barrier permeability and disturb the reverse cholesterol transport among others, affecting therefore the susceptibility for certain metabolic disorders such as obesity, diabetes or alcoholic fatty liver disease^15–21^. However, despite all these great advances in the knowledge of host physiology microbiota involvement, there are still few studies in humans revealing both the identity of microbes and their metabolites associated with host lipid metabolism.

The aim of this study was to compare both the faecal microbiota composition and the SCFAs and BA profile of subjects with hypercholesterolemia (HC) and normocholesterolemia (NC) in order to determine whether hypercholesterolemia is associated with a particular faecal bacteria signature. In addition, correlations between these both parameters and a wide selection of lipid profile biomarkers, taking into account their particle size, were analysed.

## Results

### Serum lipid profile characterization of HC and NC

HC group displayed the typical hallmarks of this phenotype in comparison with NC group **(**Table 1**)**, such as higher Total-C levels, mainly carried by intermediate-density lipoprotein (IDL-C) and LDL (LDL-C), higher levels of triglycerides (TG) from IDL and LDL, as well as higher ratio of both Total-C to HDL-C and LDL-P to HDL-P. With regard to the amount of lipoprotein particles classified accordingly their size, HC showed higher levels of both large, medium and small LDL particles (large-LDL-P, medium-LDL-P and small-LDL-P), although its average size (LDL-Z) were similar in both groups. Respect to HDL, the amounts of small particles (small-HDL-P) was also increased in HC, whereas the average diameter (HDL-Z) was slightly higher in NC. Nevertheless, APOA1 levels, the major component of HDL in plasma, did not present significant differences between both populations.

**Table 1:** Demographic, anthropometric and biochemical features of HC and NC subjects.

	HC	NC	p-value	q-value
**Demographic features**				
N	21	9		
Sex	Male	Male		
Race	Caucasian	Caucasian		
Country	Spain	Spain		
Region	Catalonia	Catalonia		
Age (years)	54.5 ± 7.5	43.67 ± 7.48	0.0024	**0.0114**
**Anthropometric data**				
BMI (kg/m^2^)	28.4 ± 2.8	26.18 ± 5.37	0.0315	0.0883
WHR	0.96 ± 0.04	0.91 ± 0.04	0.0080	**0.0281**
Body fat percentage	26 ± 4.7	25.2 ± 6.7	0.7483	0.7483
Biceps fold (mm)	8.0 ± 2.3	7.26 ± 3.41	0.2264	0.3169
Triceps fold (mm)	14.6 ± 4.7	15.5 ± 7.2	0.6248	0.6248
Subscapular fold (mm)	22.3 ± 6.4	21.17 ± 8.5	0.7182	0.7483
Suprailiac fold (mm)	15.5 ± 5.4	11.4 ± 4.7	0.0563	0.0985
**Blood pressure (mmHg)**				
Systolic blood pressure	134.4 ± 17.4	119.56 ± 12.21	0.0401	0.0899
Diastolic blood pressure	88.4 ± 11.2	80.89 ± 9.92	0.0945	0.1469
Mean blood pressure	106.1 ± 27.6	100.22 ± 10.4	0.0449	0.0899
**Blood biochemistry**				
Glucose (mg/dL)	109.22 ± 9.78	97.6 ± 7.0	0.0025	**0.0175**
Lactate (mg/dL)	26.6 ± 9.5	22.2 ± 11.8	0.3444	0.4822
Uric acid (mg/dL)	5.9 ± 1.4	4.4 ± 0.4	0.0002	**0.0024**
**Lipids**				
*Cholesterol (C) (mg/dL)*				
VLDL-C	16.93 ± 11.23	8.61 ± 4.35	0.0503	0.0795
IDL-C	10.77 ± 4.28	6.8 ± 2.35	0.0022	**0.0055**
LDL-C	123.32 ± 22.08	89.48 ± 16.73	<0.0001	**0.0003**
HDL-C	51.61 ± 7.59	52.55 ± 9.13	0.8994	0.8994
Total-C	202.62 ± 30.32	157.44 ± 24.14	0.0002	**0.0009**
*Triglycerides (TG) (mg/dL)*				
VLDL-TG	88.34 ± 58.2	47.06 ± 18.97	0.0503	0.0795
IDL-TG	11.7 ± 3.47	8.31 ± 1.99	0.0027	**0.0063**
LDL-TG	15.4 ± 4.78	9.57 ± 3.04	0.0003	**0.0008**
HDL-TG	14.67 ± 4.91	12.32 ± 2.38	0.3722	0.4136
Total-TG	130.12 ± 64.17	77.26 ± 21.46	0.0315	0.0676
*Particle number (P)*				
VLDL-P (nmol/L)	58.59 ± 40.55	30.35 ± 12.52	0.0563	0.0844
Large VLDL-P (nmol/L)	1.8 ± 0.9	1.11 ± 0.5	0.0449	0.0795
Medium VLDL-P (nmol/L)	8.7 ± 4.99	5.01 ± 1.75	0.0111	**0.0208**
Small VLDL-P (nmol/L)	48.09 ± 34.96	24.23 ± 10.36	0.0627	0.0896
LDL-P (nmol/L)	891.61 ± 161.53	637.23 ± 119.17	<0.0001	**0.0003**
Large LDL-P (nmol/L)	108.36 ± 24.97	79.65 ± 16.69	0.0005	**0.0014**
Medium LDL-P (nmol/L)	324.09 ± 72.68	230.39 ± 53.33	0.0006	**0.0017**
Small LDL-P (nmol/L)	459.16 ± 81.6	327.19 ± 58.95	<0.0001	**0.0003**
HDL-P (μmol/L)	29.14 ± 4.13	27.13 ± 3.33	0.2022	0.2426
Large HDL-P (μmol/L)	0.19 ± 0.06	0.16 ± 0.05	0.2452	0.2829
Medium HDL-P (μmol/L)	7.41 ± 2.25	8.51 ± 1.88	0.3375	0.375
Small HDL-P (μmol/L)	21.54 ± 3.93	18.46 ± 2.1	0.02424	**0.03463**
*Particle Diameter (Z)*				
VLDL-Z (nm)	42.55 ± 0.56	42.8 ± 0.36	0.1623	0.2029
LDL-Z (nm)	21 ± 0.2	21.02 ± 0.16	0.7558	0.7819
HDL-Z (nm)	8.17 ± 0.06	8.22 ± 0.04	0.0387	0.05279
*Other Parameters*				
Non-HDL-P (nmol/L)	921.06 ± 167.78	640.45 ± 112.93	<0.0001	**0.0001**
Total-P/HDL-P	32.92 ± 5.72	24.59 ± 2.94	<0.0001	**0.0001**
LDL-P/HDL-P	30.93 ± 5.72	23.44 ± 3.06	<0.0001	**0.0001**
Total-C/HDL-C	3.97 ± 0.64	3.02 ± 0.3	<0.0001	**0.0001**
ApoA1 (mg/dL)	136.68 ± 22.07	142.49 ± 22.84	0.5291	0.5669

Mean and standard deviation of each numeric variable are shown. p-value and q-value obtained after adjustment for multiple testing are shown. q-values < 0.05 are indicated in bold. Abbreviations used: BMI (body mass index), WHR (waist to hip ratio), very low density lipoprotein (VLDL), intermediate density lipoprotein (IDL), low density lipoprotein (LDL), high density lipoprotein (HDL).

### Diversity of faecal bacterial species in HC and NC

**Figure 1:**
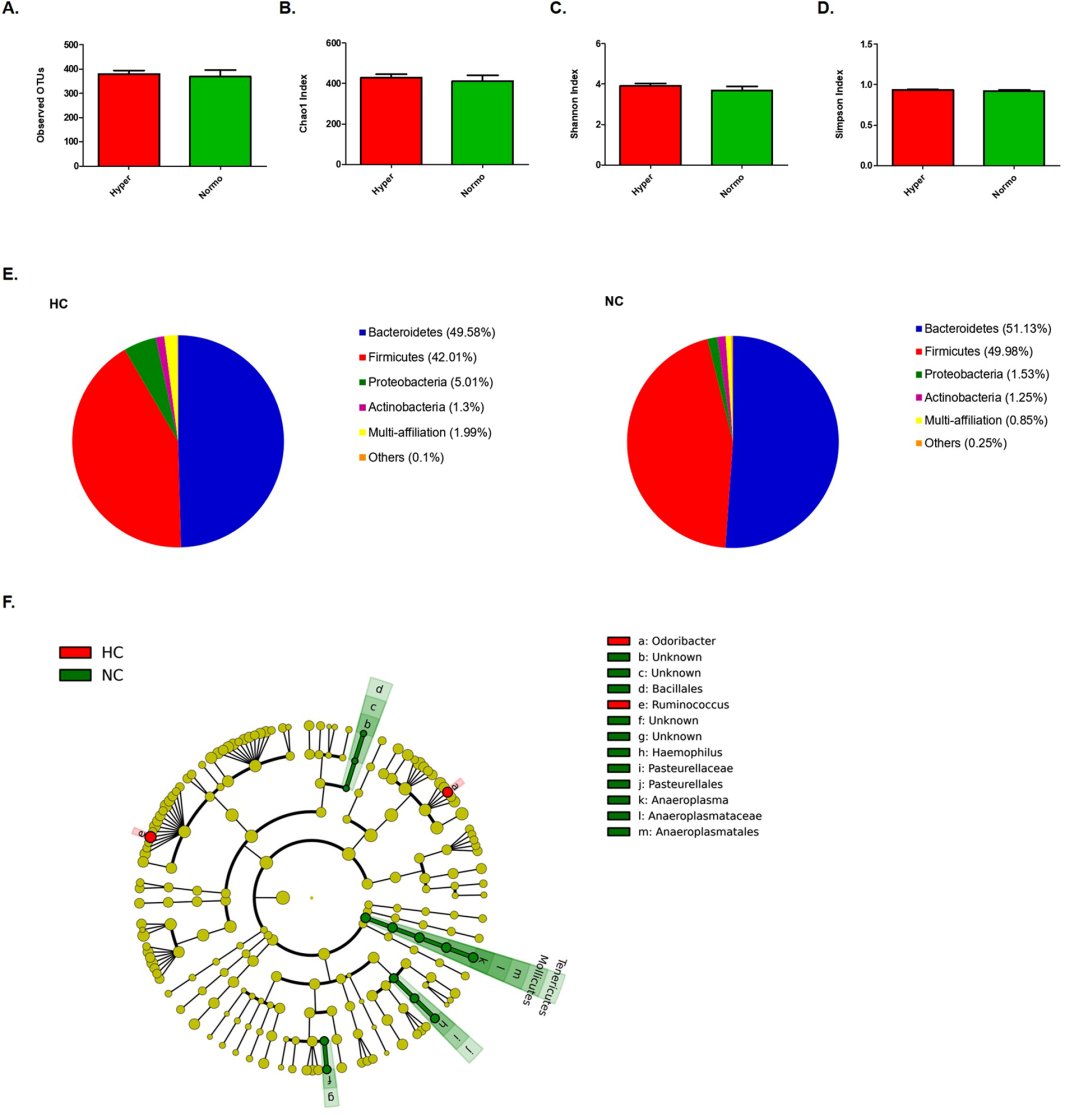
Species diversity and faecal bacterial taxonomic signature in HC versus NC subjects. Diversity indexes: (**A**–**D**) Observed OTUs, Chao1, Shannon’s and Simpson’s indexes, respectively. (**E**) Pie chart of phyla relative abundances identified in HC and NC, respectively. (**F**) Cladogram plot of discriminant taxa identified by LEfSe analysis, p-value < 0.05 as significant.

The assessment of bacterial species diversity did not display relevant differences between HC and NC subjects (Fig. 1A–D). Thus, the number of observed operational taxonomic units (OTUs) (HC, 380.1; NC, 368.3; p-value = 0.79) as well as the average of Chao’s index (HC, 430.1 and NC, 412.1; p-value = 0.55) displayed analogous values in HC and NC subjects. Likewise, Shannon’s and Simpson’s indexes revealed a similar diversity in both groups (Shannon’s Index: HC, 3.92 and NC, 3.69, p-value = 0.37; Simpson’s Index: HC, 0.94 and NC, 0.92, p-value = 0.32).

### Taxonomic characterization of the faecal bacterial community in HC and NC

Taxonomic characterization analysis revealed some differences between the faecal bacterial communities of HC and NC subjects (Fig. 1E–F, Supplementary Tables S1, S2). Particularly, at phylum level, these differences were observed among the tail of rare bacteria, since HC displayed less relative abundance of *Tenericutes* than NC (0.002% vs 0.22%, respectively; p-value = 0.026, q-value = 0.2812). This differential taxonomic feature was maintained from phylum to genus level, thus HC showed reduced abundance of *Mollicutes* (Class), *Anaeroplasmatales* (Order), *Anaeroplasmataceae* (Family) and *Anaeroplasma* (0.002% vs 0.22%, p-value = 0.026; q-value = 0.8573).

No differences were observed in dominant phyla such as *Bacteroidetes* or *Firmicutes* (HC, 49.58% vs NC, 51.13% and HC, 42.01% vs NC, 49.98%, respectively), and either in *Proteobacteria* (HC, 5.01% vs NC, 1.53%, respectively) (Fig. 1E). The ratio *Firmicutes* to *Bacteroidetes*, which has been reported to change through age^22^ and in certain metabolic pathologies such as obesity^23^, was also similar in both groups (data not shown). Nevertheless, there were three bacterial genera belonging to these phyla which were found to have different prevalence in both groups: *Odoribacter* (*Bacteroidetes*), *Ruminoccocus* (*Firmicutes*) and *Haemophilus* (*Proteobacteria*), (Fig. 1F, Supplementary Tables S1, S2). *Haempohilus* displayed lower relative abundance in HC (HC, 0.04% vs NC, 0.08%, p-value = 0.049, q-value = 0.8573), whereas in contrary, the others showed higher relative abundance in these subjects (*Odoribacter*, 0.51% vs 0.16% p-value = 0.044, q-value = 0.8573; *Ruminococcus*, 2.33% vs 1.12% p-value = 0.049, 0.8573). Only two species belonging to *Phascolarctobacterium* genus were observed differently represented in both groups (Supplementary Table S1), displaying less relative abundance in HC than in NC: *P*. *faecium* (1.32% vs 2.26%, p = 0.042, q-value = 0.764) and *P*. *succinatutens YIT 12607* (1.18% vs 2.62, p-value = 0.046, q-value = 0.764).

### Influence of diet and age over the observed faecal bacterial community composition in HC and NC

Diet is a key factor in gut microbiota shaping^24^. Therefore, its potential influence over the differences observed in the faecal bacterial composition between HC and NC subjects was studied. No differences were observed in the studied dietary parameters between both groups (Supplementary Table S3). Nevertheless, it was observed that *Haemophilus* correlated positively to saturated fatty acids levels (SFA), *Odoribacte*r did negatively with both dietary energy and carbohydrates intake, and in the case of *Ruminococcus*, it correlated negatively to energy intake, fat, SFA and monounsaturated fatty acids (MFA). *Anaeroplasma* was not associated with any dietary parameter (Supplementary Fig. S1).

Age is another key factor in microbiota modulation. Particularly it has been shown that diversity changes through the different stages of life, remain almost stable during adulthood until old age^25^. In the present study, although age was significantly different between HC and NC when compared by Student-t test (Table 1), both groups were in the same range of age (adulthood), which was reflected by a similar faecal bacteria diversity. Nevertheless, with regard to the taxonomical differences observed, associations analysis revealed that the age correlated positively with *Ruminococcus* relative abundance (*rho*: 0.543, p-value = 0.002) (Supplementary Fig. S2), but not with the other genera (data not shown).

### Correlation between faecal bacterial community and serum lipid profile

**Figure 2:**
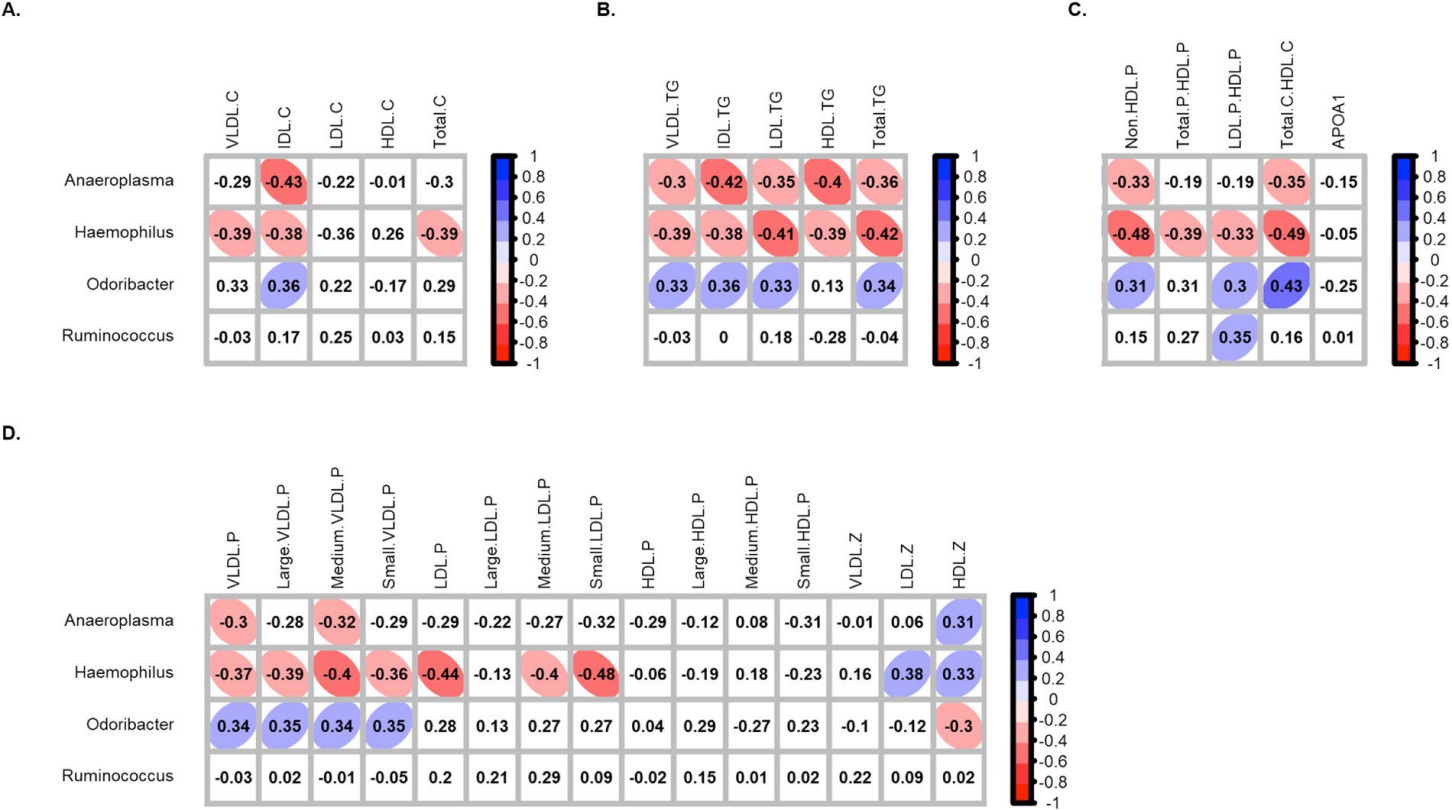
Correlation between serum lipid biomarkers and differential genera in HC and NC subjects. Correlation with (**A**) cholesterol-related biomarkers, (**B**) triglyceride-related biomarkers, (**C**) lipoprotein ratios and APOA-1 and (**D**) lipoprotein particles classified according their size. Correlations were assessed considering data from both groups together. Correlations with q-values below 0.15 after adjustment for multiple analysis are highlighted with an ellipse. The colour and slope of the ellipse indicate magnitude of correlation, with Spearman’s *rho* value superimposed on the ellipse. The ellipses of positive correlations are shown in blue and the negative correlations in red. Correlations with q-values > 0.15 are in white.

In order to determine if there was some kind of association between the lipid profile and the faecal bacterial composition of the studied subjects, correlation analysis between both parameters, taking into account the four genera differently represented in HC and NC, were assessed by Spearman’s correlation method. As result, after correction for multiple testing; *Aneroplasma* (*Tenericutes* phylum), displayed a negative correlation with IDL-C, all biomarkers TG-related, total and medium very low density lipoproteins (VLDL) and the ratio Total-C to HDL-C (Fig. 2, Table S4). *Haemophilus* (*Proteobacteria* phylum) displayed a negative correlation to the same biomarkers, and also with Total-C, all biomarkers related to VLDL, and the total amount of non-HDL-P and LDL-P, particularly, the medium and small-LDL-P, among others. In contrary, *Odoribacter* (*Bacteroidetes* phylum) displayed a positive correlation with almost all biomarkers mentioned above, including the ratio Total-C to HDL-C. Regarding the average size of lipoproteins, *Odoribacter* correlated negatively to HDL-Z, whereas *Anaeroplasma* and *Haempohilus* did positively. In the case of *Ruminococcus* (*Firmicutes* phylum), no significant correlations were observed.

Respect to the remaining genera identified in the studied subjects, *Enterorhabdus* and *Turicibacter* were the genera which showed correlation with more lipid biomarkers (Supplementary Fig. S3). Thus, *Enterorhabdus* displayed positive correlations with almost all VLDL related biomarkers and Total-TG, but negative to HDL-Z; whereas *Turicibacter* correlated negatively to IDL-TG, HDL-TG, Total-TG and with all VLDL-related biomarkers, but positive to HDL-Z.

### Faecal bacterial co-occurrence

The effect of microbiota on human metabolism depends on both synergist and antagonist effects between the present microbes of the community rather than to the behaviour of a particular microbe. Therefore, co-occurrence between discriminant bacteria genera in HC and C and the remaining bacteria was also study (Table S5). *Anaeroplasma* showed correlations with seven genera, among which *Acetivibrio*, *Desulfovibrio*, *Oxolobacter* and *Roseburia* correlated also with lipid profile. *Haemophilus*, it was found to correlate with *Desulfovibrio*, *Slackia* and *Veillonella* among others. *Odoribacter* abundance seemed to be associated with six genera, among which were *Ruminococcus*, the genus associated with more bacteria.

### Bacterial fermentation products profile characterization and correlation with lipid profile

SCFAs are secondary metabolites resulting from bacterial action on diet indigestible fraction that may influence host metabolism^26^. Thus, a faecal dysbiosis may involve an alteration in SCFAs profile that could alter the host lipid metabolism. Therefore, the levels of most common SCFAs present in faeces (acetic, propionic and butyric acid), as well as isobutyric and isovaleric acids, were assessed in faecal and serum samples. Succinic acid levels were also analysed in faeces, since it is an organic acid commonly present as result of bacterial activity.

**Figure 3:**
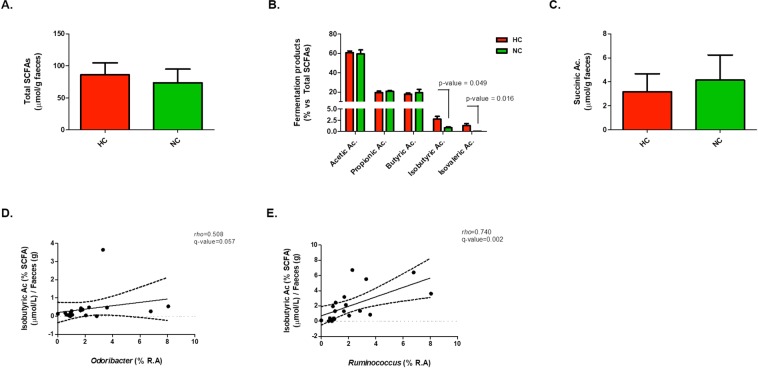
Faecal bacterial fermentation products profile in HC and NC subjects and significant correlation with bacterial abundance at genus level. (**A**–**C**) Total SCFAs, SCFAs profile and succinic acid levels, respectively. P-value < 0.05 after comparison between HC and NC are indicated. (**D**,**E**) Correlation plots of isobutyric acid with the genera *Odoribacter* and *Ruminococcus*, respectively. Spearman’s *rho* and q-value are shown.

The results from the faecal samples revealed that the total SCFAs and succinic acid levels were similar in HC and NC (Fig. 3A,C). However, in terms of proportions, both isobutyric (2.76% vs 0.82%, p-value = 0.049) and isovaleric acid (1.32% vs 0.06%, p-value = 0.016) displayed higher abundance in HC than NC (Fig. 3B), whereas acetic, propionic and butyric acids levels were not different between both groups.

**Figure 4:**
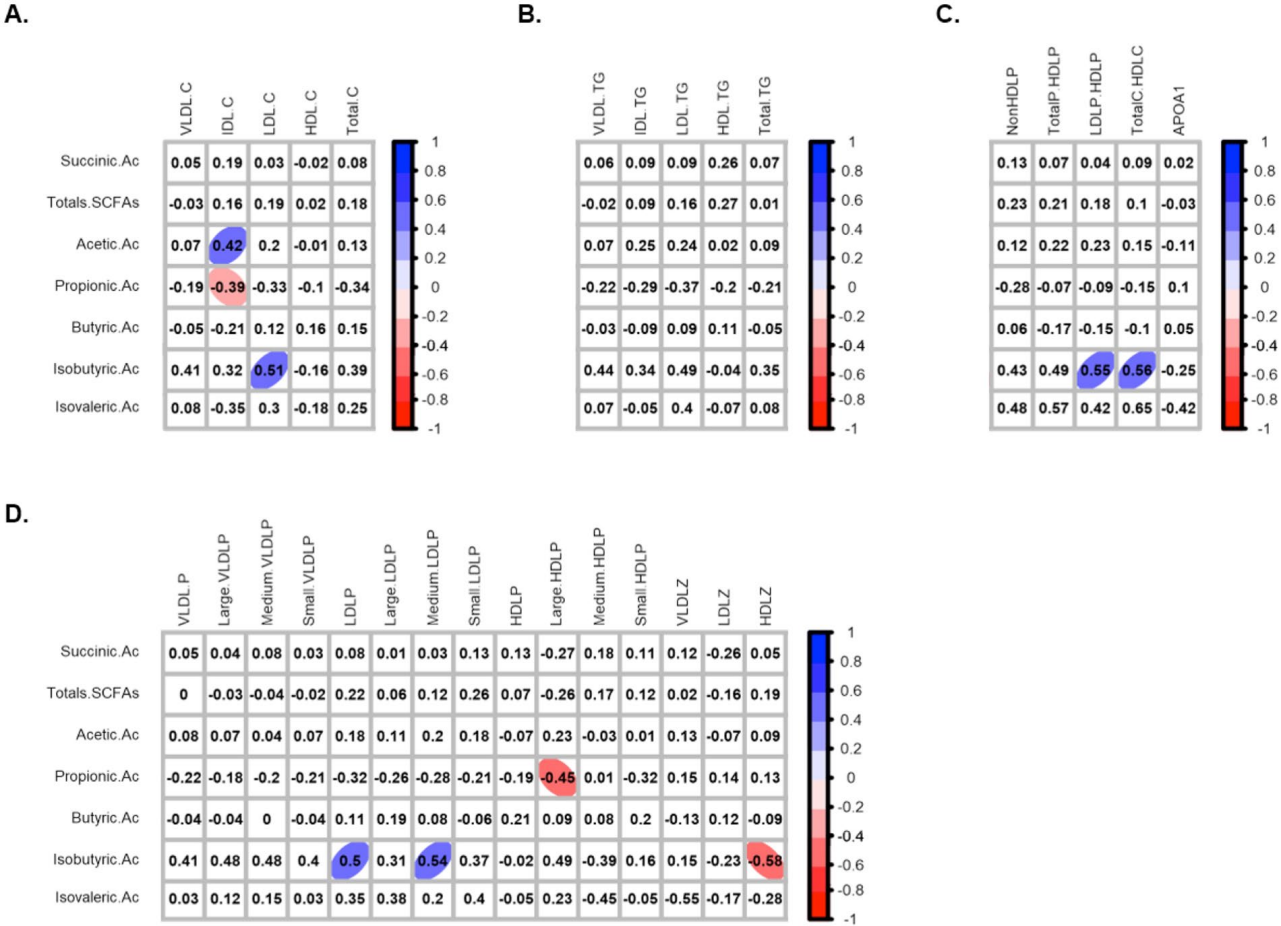
Faecal bacterial fermentation products profile and lipid biomarkers association in HC and NC subjects. Correlation with cholesterol-related biomarkers (**A**), triglyceride-related biomarkers (**B**), lipoprotein ratios and APOA-1 (**C**), and lipoprotein particles classified according their size (**D**). Correlations were assessed considering data from both groups together. Correlations with q-values below 0.15 after adjustment for multiple analysis are highlighted with an ellipse. The colour and slope of the ellipse indicate magnitude of correlation, with Spearman’s *rho* value superimposed on the ellipse. The ellipses of positive correlations are shown in blue and the negative correlations in red. Correlations with q-values > 0.15 are in white.

In order to study whether the higher proportion of both isobutyric and isovaleric acid in faeces from HC versus NC was related to the differences observed in bacteria composition and lipid profile, correlation analysis between them were performed. Results revealed that the isobutyric acid proportion was positively associated with *Odoribacter* and *Ruminococcus* abundances **(**Fig. 3D–E). Isovaleric acid did not show association with these genera. In relation to blood lipid profile, isobutyric acid seemed to correlate positively with LDL-C, LDL-P, medium-LDL-P and the ratio LDL-P/HDL-P, and negatively with HDL-Z **(**Fig. 4A–D, Table S6). With respect to the other SCFA, it was observed a positive correlation between acetic acid and IDL-C, whereas propionic acid correlated negatively to this last biomarker and also with the large HDL-P levels **(**Fig. 4A–D, Table S6). No correlations were observed with butyric and isovaleric acids.

Serum SCFAs profile characterization revealed that, as well as in faeces, the total SCFAs levels were similar in HC and NC (Supplementary Fig. S4A). Among the SCFAs detected, only butyric acid showed different levels in HC and NC, being more abundant in the latter (15.0 vs 19.0 µmol/L, p-value = 0.0021) (Supplementary Fig. S4B). Isovaleric acid was not detected. Correlation analysis revealed that serum levels of butyric acid correlated negatively to several lipid biomarkers, most of them related to low density lipoproteins levels such as LDL-C, LDL-TG, large and small-LDL-P, as well as Total-C and Total-C to HDL-C ratio among others (Supplementary Fig. S4C). Serum levels of acetic acid and the total amount of SCFAs correlated positively to APOA-1 levels, whereas isobutyric levels were observed to correlated negatively to LDL-C. No correlations with propionic acids levels were observed.

Other metabolites from bacteria activity in the gut are the secondary products from cholesterol and BA^18^, so thus their abundance and composition are linked to bacteria community presence and, presumably, its composition. According to this and due to their relation to the host lipid profile, the abundance of these metabolites in the faeces of HC and NC, as well as their potential associations with the discriminant genera observed among both groups, was assessed. The results revealed that total levels of sterols in faeces of HC and NC were similar (Supplementary Fig. S5A). Specifically, both cholesterol and their derived metabolites showed similar values in both groups. Likewise, the analysis of BA profile revealed that the amount of chenodeoxycholic acid (CDCA), cholic acid (CA), lithocholic acid (LCA) and deoxycholic acids (DCA) were similar in the faeces of HC and NC (Supplementary Fig. S5B). Correlation analysis revealed some associations between the discriminant genera and these metabolites. Thus, *Haemophilus* was observed to be negatively associated with the levels of total sterols, coprostanol and DCA, whereas *Odoribacter* and *Ruminococcus* were both negatively associated with cholesterol levels and positively to coprostanol, and in case of the latter, cholestanol too (Supplementary Fig. S5C). *Anaeroplasma*, however, did not show correlation with sterols or BA.

## Discussion

Gut microbiota has been suggested to play an important role in host’s metabolism, including lipid levels modulation^20,27^. One of the main contributions of the present work to this issue is the proposal of a specific faecal bacteria signature in individuals with hypercholesterolemia, characterized by lower relative abundance of both *Anaeroplasma* (*Tenericutes*) and *Haemophilus (Proteobacteria*) and higher relative abundance of both *Odoribacter* (*Bacteroidetes*) and *Ruminococcus (Firmicutes)* compared with normomocholesterolemic subjects. These differences seem to be intrinsically related to the subject physiology rather than to other external factors that have been reported to alter the gut microbiota composition, such as diet; since although some association were observed between both carbohydrates, fat, MFA and SFA with *Haemophilus*, *Odoribacter* and *Ruminococcus*, the intake of these nutrients was similar in HC and NC. Only in the case of *Ruminococcus*, the differences observed were associated with age, so that further studies in a large cohort would be needed to confirm or discard its contribution to the faecal bacterial signature in this pathology.

In another context, *Tenericutes* and *Odoribacter* seem to have a high heritability, as has been recently reported in a study performed on monozygotic and dizygotic twins and their families^28^. Their alterations have been also described in other metabolism-related pathologies such as obesity, metabolic syndrome and diabetes^21,29^, suggesting that the dysbiosis of these bacteria in HC subjects could have a certain hereditary component which is common to other metabolic disorders as well. In fact, patients with phenylketonuria, an inborn metabolic error, have been reported to have lower *Odoribacter* and *Ruminococcus* abundance^30^ and interestingly, a study addressed by Moseley revealed that almost 26% of phenylketonuria patients had blood lipid profile alteration^31^. In the case of *Haemophilus*, a genus which includes some species related to pathogenicity, it is unknown which factors may modulate their population, although its decline has been also reported in other metabolic disorders such type II diabetes^32^.

Furthermore, correlation analysis with serum lipid biomarkers support and stand out the relevance of these bacteria in hypercholesterolemia. Particularly, in the case of *Haemophilus*, a negative correlation with a common indicator of dyslipidaemia such as the Total-C to HDL-C ratio was observed, but also with TG and novel biomarkers related to lipoprotein size, such as the average size of HDL, or the amount of small-LDL-P, which has been recently reported to be associated with a higher incidence of peripheral artery disease in women^33^. These findings are consistent with results recently reported by Fu and colleagues, in which bacteria of the family to which this genus also belongs (*Pasteurellaceae*) showed a negative correlation to TG levels^27^. Similarly, it was observed that the lower abundance of *Anaeroplasma*, was associated with an unfavourable lipid profile (IDL-C, TG-related biomarkers and the ratio Total-C to HDL-C among others). Previous studies had already reported an association between both TG and HDL levels with *Tenericutes* phylum, but until now it was unknown which bacteria from this phylum showed this sort of correlation^34^. With regard to *Odoribacter*, the lower abundance, the better lipid profile was observed, since it displayed a positive correlation to both IDL-C and IDL-TG, Total-TG, VLDL-P and the ratio of Total-C to HDL-C, among others. These results are consistent with previous data of Fu and colleagues, suggesting a negative correlation of this genus to HDL-C^27^, as well as a positive association with other related metabolic parameters such as the body fat percentage, adiposity index and visceral fat^29^.

Moreover, another interesting point that highlights the relevance of these four genera in dyslipidaemia is the fact that they seem to influence on the abundance of other bacteria such as *Slackia*, *Desulfovibrio*, *Oxalobacter*, *Roseburia*, *Oscillibacter*, *Parabacteroides and Turicibacter*; which according to results from this study are also associated with blood lipid levels.

At species level, HC seem to have a lower relative abundance in *Phascolarctobacterium faecium* and *Phascholarctobacterium succinatutens YIT12067*. Both are bacteria that mainly use succinate as energy source^35^, which suggests that differences in this energy source could lead to differences in their abundances. However, in the present work, the levels of succinic acid in faeces were similar in HC and NC subjects. *P*. *succinatutens* has been described as a kind of bacteria unable to grow in presence of bile acids^35^, so that its lower abundance in HC individuals could be associated with an increase of faecal bile acids. Nevertheless, results of this work revealed that the amount of bile acids in the faeces of HC and NC were similar.

One of the main mechanism by which gut microbiota may influence on host physiology is linked to their metabolic activity^26,36^. The major end products of bacterial fermentation in colon are organic acids such as acetate, propionate, butyrate, lactate and succinate, together with hydrogen, methane and CO_2_^37^. Mounting evidences suggest that SCFAs have a high impact on host physiology since they can act as energy source for epithelial cells, signalling molecules and gene expression modulator^26,36,38^. The amount and relative abundance of each SCFA depend on diet’s indigestible fraction^24,38^, but also on a cross-feeding mechanisms established in the bacterial community^39,40^. The most abundant SCFAs are acetic acid (C2), propionic acid (C3) and butyric acid (C4), which together represent nearly 90–95% of the SCFA present in the colon^41^. Acetate is a net product of carbohydrate fermentation of most anaerobes bacteria, while propionic and butyric acid are generated from carbohydrate or protein fermentation by a distinct subset of bacteria^42^. In the present study, both faecal and serum total SCFAs were similar in HC and NC. Nevertheless, data from correlation analysis revealed that the faecal proportion of acetic acid correlated positively to IDL-C levels, whose high levels are indicative of a more unfavourable lipid profile, while propionic acid did negatively. The serum levels of acetic and propionic acids, although were not significantly different between groups, displayed a profile characterized by higher and lower abundance of acetic and propionic acids, respectively, in HC than NC. These findings are consistent with data from previous studies showing that circulating acetic acid is related to “*de novo*” lipogenesis and cholesterogenesis stimulation in the liver, while propionic acid seemed to inhibit them^43,44^, which reinforced the hypothesis that the ratio of acetic acid to propionic acid could be considered as a novel biomarker of the host lipid profile status. Nevertheless, with regard to the role of acetic acid in lipid metabolism there are some controversies, since other authors have reported that dietary acetic acid is related to a decrease of both cholesterol and triglyceride levels in rats^45^. Abundance of butyrate in faeces did not show difference between HC and NC and showed no correlation with any of the analysed lipid biomarkers in the present study. However, its serum levels were higher in NC, revealing a negative correlation to lipids related to a worst profile, such as LDL-C, Total-C, LDL-TG, LDL-P (large and small) and Total-C to HDL-C ratio among others. Nevertheless, previous studies have reported that butyrate stimulates fatty acid synthesis and cholesterogenesis^46^. Contrary, an interesting paper published by Gao Z *et al*. reported that supplementation of butyrate in diet is able to prevent other metabolic pathologies such as insulin resistance in rat, associated to a mechanism of energy expenditure and induction of mitochondrial function^47^.

The last relevant finding from this study revealed the role of isobutyric and isovaleric acids in human lipid metabolism. Both are considered branched short chain fatty acids (BSCFAs). They can be generated from valine and leucine fermentation^26,48–50^ and only contribute to 5% of the total SCFAs production. Data from the present work, revealed that the proportion of both BSCFAs seemed to be higher in HC than in NC, suggesting an increase of amino-acid fermentation in this group which in turn, strengthens the accumulation of other potentially harmful metabolites such as indole, amines, *p*-cresol or ammonia among others^51,52^. According to this, in the present study, isobutyric acid was observed to be associated with a more unfavourable lipid profile, since the higher levels of this BSCFA, the higher values of LDL-C, total LDL-P, medium-LDL-P, LDL-P/HDL-P ratio, Total-C/HDL-C ratio and lower HDL-Z were observed. These data are consistent with previous studies reporting cAMP-mediated lipolysis inhibition and insulin-stimulated de novo lipogenesis in primary rat adipocytes by BSCFA^53^. The differential production in BSCFA could be explained by the difference in bacterial relative abundance. Most of proteolytic bacteria reported in literature belong to *Bacteroides* and *Clostridium* genera^37^. However, none of them was differently represented in HC and NC. Instead, the genera *Odoribacter*, from which some species have been related to isovaleric acid production^54^, and *Ruminococcus*, which has been reported to required BSCFA for growth^55,56^ were found to be more abundant in HC than in NC, and both showed a positive correlation with isobutyric proportions observed in faeces.

The activity of faecal bacteria is also related to both bile acids and cholesterol metabolization, whose derived products may influence host lipid metabolism^18^. Results from this work revealed that *Odoribacter* and *Ruminococcus* were negatively associated with faecal cholesterol levels and positively to coprostanol and cholestanol, that might indicate a higher metabolism of this sterol in HC. However, the similarity in the abundance of coprostanol and cholestanol in the faeces of HC and NC ruled this hypothesis out. Likewise, *Haemophilus* displayed a negative association with DCA, but the levels of the bile acids were similar in both groups, discarding that the relation between the lipid profile and the discriminant bacteria observed in both groups was due to these metabolites.

### Remarkable conclusions

This study revealed that individuals with hypercholesterolemia possess a particular faecal bacterial signature, characterized by lower prevalence of the genera *Anaeroplasma* and *Haemophilus* and higher prevalence of *Odoribacter*, which seems to be associated with a wide range of blood lipid biomarkers, including those ones commonly linked to a higher risk of CVDs and others considered as novel promising biomarkers related to both amount and size of lipoproteins. Furthermore, it was observed in HC subjects a raise in isobutyric acid, whose higher abundance in the faeces could be considered as a biomarker in hypercholesterolemia.

The lower prevalence of both *P faecium* and *P Succinatutens* in the faeces of hyperhcolesterolemic subjects is suggested as another hallmark of the bacterial community dysbiosis associated with higher cholesterol levels but its involvement in the lipid profile is still unclear.

Nonetheless, in spite of the relevant findings here shown, the authors assume that the study has some limitations. It was performed only in men, all of them from a specific area of Spain (Catalonia), the number of subjects was low and moreover, the number of healthy volunteers was lower than the hypercholesterolemic ones. Methodologically, 16S rRNA sequencing data has been a revolutionary technique in last years, but it has some limitations related to detection at species level with respect other methods more expensive currently used. To confirm these results, mainly descriptive, further studies addressed to perform microbiota colonization of germ-free mice through the administration of faeces from volunteers with normo- and hypercholesterolemia would be appropriated.

## Methods

### Subjects and study design

**Figure 5:**
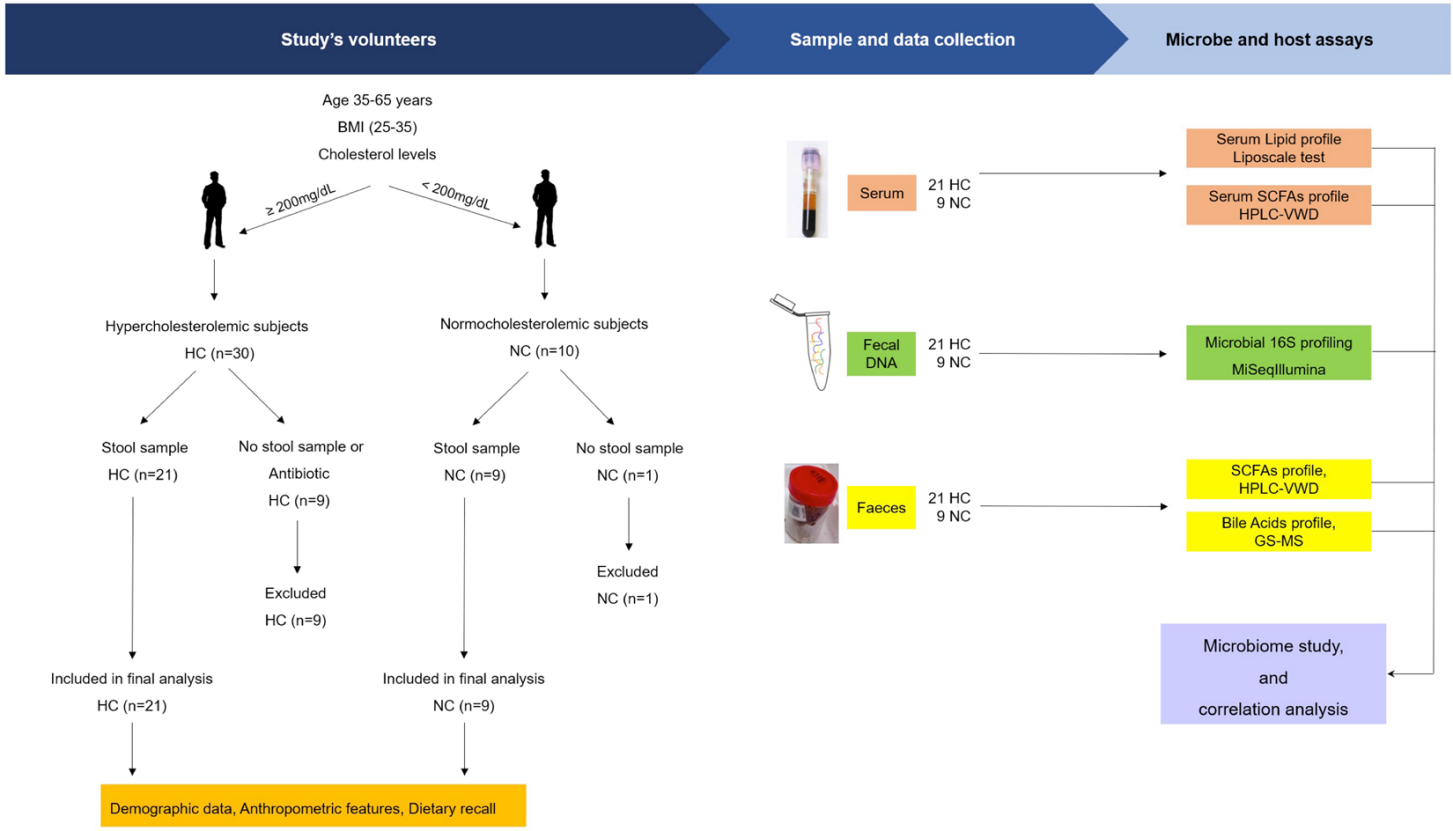
Study design flowchart.

In total, 30 men aged between 35–65 years from Lleida (Spain), whose demographic data are provided in Table 1, participated in the study. Due to the reported influence of androgens on the lipid profile, women were not included in this study. Volunteers with known history of hypercholesterolemia were recruited from Primary Care Centres in Catalonia, with the collaboration of Centre’s Family Physicians. More than 3 blood test analyses that revealed high levels of total cholesterol (>200 mg/dL) was used as defining criteria for the hypercholesterolemic phenotype. The exclusion criteria included a body mass index (BMI) out of the range 25–35 kg/m^2^, suffering metabolic pathologies such as type 1 or type 2 diabetes and having taken lipid-lowering medications, probiotic supplements and/or antibiotics in last two months. Volunteers with no history of high levels of total cholesterol (<200 mg/dL) were selected as control group. All volunteers were adequately informed before giving their consent and no one of them have taken any medications. The study, whose experimental design is shown in Fig. 5, was approved by the clinical research ethical committee of the Institut Catalá de Salut from Hospital Universitary Arnau de Vilanova, in Lleida, Spain (CEIC-1534. 21/12/2015) and it was conducted according to the ethical guidelines of the Helsinki Declaration.

### Blood biochemistry

Venous blood samples from each volunteer in fasting conditions (12 hours) were collected in serum separator tubes (BD, Ref. 367953) and centrifuged immediately for 15 min at 4 °C at 1500 g. Then, the serum was collected and kept at −80 °C until the further biochemical analysis. Total-C and TG levels, as well as their content in lipoproteins (VLDL, IDL, HDL, LDL) and the average size of each particle were measured through Liposcale test by the Research Unit on Lipids and Atherosclerosis of Sant Joan University Hospital and Universitat Rovira i Virgili, at Reus (Spain), according to the procedures described by Mallol and colleagues^57^. APOA1 levels as well as glucose, lactate and uric acid were quantified by SPINREACT kits following manufacturer’s instructions (Ref. 93010, 1001010, 1001190, 1001330, respectively).

### Microbial DNA purification, 16S amplicon preparation, sequencing and processing

In order to characterize the faecal bacterial community of both hypercholesterolemic and healthy volunteers, they were asked for a single stool sample produced at any time of the day before to the blood extraction, with no specific dietary restrictions, which were collected in sterile plastic containers (Deltalab, Ref. 4097226) and kept at −20 °C until their delivery the day after. Once in the laboratory, samples were divided in aliquots of approximately 180–220 mg and frozen at −80 °C until further analysis. Microbial DNA was extracted from the stool samples using the QIAamp DNA Stool Mini Kit (Qiagen, Hilden, Germany, Ref. 51504) as described per manufacturer’s instructions with a slight modification which included a previous bead-beating step (1 cycle of 40 seconds at 4 °C) in Lysing Matrix E tubes for FastPrep 24 (MP Biomedicals, 11452420). DNA quality and yield were evaluated via 1% (w/w) agarose gel electrophoresis and NanoDrop 2000 UV spectrophotometer (Thermo Fisher Scientific, Waltham, MA, USA).

The 16S rRNA gene libraries were generated by the company Vaiomer in Toulouse (France). Briefly, bacterial populations were determined using next generation high throughput sequencing of variable regions of the 16S rDNA bacterial gene using 16S universal primers targeting the V3-V4 region of the bacterial 16S ribosomal gene. The joint pair length was set to encompass 476 base pair amplicon thanks to 2 × 300 paired-end MiSeq kit V3. For each sample, a sequencing library was generated by addition of sequencing adapters. The detection was performed using MiSeq Illumina technology.

The targeted metagenomics sequences from microbiota were then analysed using the bioinformatics pipeline established from the FROGS v1.3.0 guidelines, and clustered into OTUs with the Swarm algorithm before taxonomic assignment as described by Lluch and colleagues^58^. Briefly, OTUs identified as chimera (with vsearch v1.9.5) in all samples in which they were present, with a relative abundance lower than 0.005% regarding the whole dataset, or with a strong similarity (coverage and identity ≥80%) with the phiX (library used as a control for Illumina sequencing runs) were removed. Clustering was produced in two passes of the swarm algorithm v2.1.6. The first pass was a clustering with an aggregation distance equal to 1 and the second one, equal to 3. The taxonomic assignment was produced by Blast+ v2.2.30+ with the databank RDP v11.4. In total, 997 OTUs were available for further analysis after filtering.

### Bacterial analysis from 16S rRNA data

#### Species diversity

Species diversity of the faecal bacteria community in each population was assessed through several indexes:(1) the number of different taxa observed (OTUs observed), which refers the actual richness observed, (2) Chao1 index, which estimates the richness of a community based upon the number of rare species that may have been missed due to under-sampling, (3) Shannon’s index, that represents the average certainty to predict the identity of unknown individuals and (4) Simpson’s index that is based upon the probability that two individuals randomly selected will belong to the same species. Results were plotted by GraphPad Prism (version 5.0).

#### Taxonomic composition of faecal microbiota profile

To statistically test for discriminant taxon between HC and NC, OTUs data from each group were compared by LEfSe (linear discriminant analysis effect size) algorithm according as described by Segata and colleagues^59^ in Galaxy/Metabiome portal, with a p-value < 0.05 as significant. Results were plot on a Cladogram. Moreover, differences in the relative abundance of each bacterial taxon (phylum, class, order, family, genus and species) were also assessed by the function runWilcox in R environment in order to obtain the p-value adjustment of each comparison for multiple testing (q-value).

### Faecal and serum short-chain fatty acids profile

The SCFAs profile present in the faeces and serum of every volunteer and succinic acid levels were determined by high-performance liquid chromatography (HPLC-VWD) according to the method previously described by Torii and colleagues^60^, where SCFAs were detected as acid hydrazides at 400 nm. Briefly, succinic and five SCFAs were assessed: acetic, propionic, butyric, isobutyric and isovaleric acids. In the extraction of SCFAs from faeces, a sample of approximately 200 mg previously frozen at −80 °C was contained in a weighed glass centrifuge tube and 5.0 mL of 70% ethanol was immediately added. The tube was weighed to determine the faecal weight, followed by mixing and centrifugation at 20 °C, 2500 rpm for 10 min. The supernatant was collected as analytical sample. Then each sample (300 µL) were derivatized with 50 µL of 2-ethylbutyric acid as internal standard and 300 µL each of pyridine, 1-EDC-HCl and 2-NPH-HCl as reaction-assistive agents, reacted at 60 °C for 20 min, and then mixed to 200 µL potassium hydroxide as reaction stopper and reacted at 60 °C for 20 min. After cooling the mixture was shaken with 3 mL of phosphoric acid aqueous solution and 4 mL of ether for 3 min for extraction and centrifuged. The obtained ether layer was shaken with 4 mL of water for 3 min and centrifuged. The ether layer was obtained, and ether was eliminated using nitrogen gas. Finally, the obtained fatty acid hydrazide was dissolved in 100 µL of methanol, and 30 µL was subjected to HPLC. All standards and reagents were purchased from Sigma-Aldrich. Analysis was performed using an HPLC of Agilent Technologies Serie 200 and a YMC-Pack FA 250 × 6 mm ID column. During the analysis, the column temperature was 50 °C; flow rate, 1.1 mL/min; and measurement wavelength 400 nm.

SCFAs levels in serum were assessed following the same protocol mentioned above and extracted with ethanol and trichloroacetic acid 5%.

### Faecal sterols and bile acids profile

Silylation of biliary acids and sterols was carried out according to Mosele and colleagues^61^, with some modifications. Briefly, 100 µL of pyridine (containing 50 µg/mL 5α-cholestan and 5β-colanic acid as internal standards) and 200 µL of N-methyl-N- (trimethylsilyl) trifluoroacetamide (both from Sigma-Aldrich) were added to vials containing 10 mg of faeces, vortexed and then maintained during 30 min at 60 °C. After silylation, samples were centrifuged 10 min at 8784 *g* at room temperature. The supernatants were analyzed by GC system (Agilent 6890 N) coupled to a mass spectrometer (Agilent 5973). Samples were injected into a capillary column DB-1MS (30 m × 0.25 mm, 0.10 μm, J&W, Agilent) and the analysis of biliary acids and sterols was performed as follows. Helium was the carrier gas (1 mL/min). Injection was carried out with a split injector (1:10) at 250 °C. The column temperature started at 240 °C and was raised at a rate of 20 °C/min until it reached 290 °C and maintained for 2 min, raised to 295 °C at a rate of 1 °C/min, and then raised at a rate of 20 °C/min to 240 °C, and maintained for 3 min (total run time 20.1 min). Peak identification was based on comparison of retention times and mass fragmentation patterns with reference compounds. All quantifications were performed in selected ion monitoring (SIM) mode using calibration curves generated from different know concentrations of commercial standards. For faecal biliary acids and sterols quantifications, commercial standards of cholic acid, deoxycholic acid, chenodeoxycholic acid, lithocholic acid, cholesterol, coprostanol and cholestanol (Sigma-Aldrich) were used.

### Food habits registration, blood pressure and anthropometric measurements

The day of blood extraction and faecal samples delivery, with the collaboration of trained dieticians, volunteers were asked for a 24-hours dietary recall of the previous day. Then, both the amount of nutrients daily ingested by each volunteer and the total energy food were estimated through DIAL software (Alceingeniería, S.A. Madrid, Spain). Additionally, blood pressure was taken using an automatic blood pressure cuff and anthropometric measurements were performed. Weight and height were measured using a portable weighting scale with an accuracy of 0.1 kg, and an incorporated steel ruler for making the size. The skinfolds thickness and estimation of total body fat were measured with a calliper, with an accuracy of 0.1 mm. The remaining anthropometric measurements were performed using a flexible measuring tape with an accuracy of 0.1 cm.

### Statistical Analysis

Statistical comparison of bacterial taxons between the two studied groups were performed by LEfSe analysis in Galaxy/Metabiome portal. Moreover, relative abundances were compared with the function runWilcox from EMA package in R environment, which adjusts the p-value for multiple testing. Diversity indexes and metadata (lipid biomarkers, anthropometric data, dietary parameters, SCFAs, sterols and BA) were compared by unpaired t-test or Wilcoxon Mann-Whitney U test in R environment, considering a p-value < 0.05 as significant. P-adjustment after false discovery rate correction from multiple comparisons was performed with runTtest or runWilcox function when more than 10 variables of the same family data set were evaluated, and q-values were added. Correlation analysis was calculated by Spearman’s correlation method in R statistical framework using the cor.test function from the stats package corrplot. P-values were adjusted for multiple comparisons. q-value considered as significant is indicated in the legend of each figure. Results were plotted in R environment using the ellipse package or with GraphPad Prism (version 5.0).

## Supplementary information

Supplementary information
