# Supplementary material for: Faecal bacterial and short-chain fatty acids signature in hypercholesterolemia
Source: Sci Rep. 2019 Feb 11;9:1772. doi: 10.1038/s41598-019-38874-3 (PMC6370822; doi:10.1038/s41598-019-38874-3)

**Supplementary information**

**Faecal bacterial and short-chain fatty acids signature in hypercholesterolemia.**

***Granado-Serrano A.B.^1^, Martín-Garí M.^1^, Sánchez V.^2^, Riart Solans M.^2^, Berdún R.^1^, Ludwig I.A.^3^, Rubió L^4^, Vilaprinyó E.^5^, Portero-Otín M.^1^ and Serrano J.C.E.^1,^****

^1^Department of Experimental Medicine, University of Lleida, Lleida, Spain

^2^Institut Català de la Salut, Catalunya, Spain

^3^Functional Nutrition, Oxidation and Cardiovascular Disease Group, Faculty of Medicine and Health Sciences, Rovira i Virgili University, Reus, Spain

^4^Department of Food Technology, XaRTA-TV, Agrotecnio Center, High Technical School of Agricultural Engineering, University of Lleida, Lleida, Spain

^5^Department of Basic Medical Sciences, University of Lleida, Lleida, Spain

* Corresponding author: [jceserrano@mex.udl.cat](mailto:jceserrano@mex.udl.cat)

**Supplementary information contains:**

- Supplementary Tables S1-S6
- Supplementary Figures S1-S5

**Table S1. Discriminant bacteria in the faeces of HC and NC subjects.** OTUs were compared by LEfSE analysis and discriminant features are shown. Class of subjects with the highest mean (HC or NC), logarithmic LDA score and p-value for each variable are indicated.

| **Discriminant bacteria** | **Class** | **Logarithmic LDA score** | **p-value** |
| --- | --- | --- | --- |
| Bacteria.Firmicutes.Negativicutes.Selenomonadales.Acidaminococcaceae.Phascolarctobacterium.Phascolarctobacteriumfaecium | NC | 3.95559E+11 | 0.0396 |
| Bacteria.Firmicutes.Negativicutes.Selenomonadales.Acidaminococcaceae.Phascolarctobacterium.PhascolarctobacteriumsuccinatutensYIT12067 | NC | 3.8444E+11 | 0.0435 |
| Bacteria.Tenericutes.Mollicutes | NC | 3.06135E+11 | 0.0238 |
| Bacteria.Tenericutes.Mollicutes.Anaeroplasmatales.Anaeroplasmataceae | NC | 3.06134E+11 | 0.0238 |
| Bacteria.Tenericutes.Mollicutes.Anaeroplasmatales.Anaeroplasmataceae.Anaeroplasma.Unknown | NC | 3.06126E+11 | 0.0238 |
| Bacteria.Tenericutes | NC | 3.0612E+11 | 0.0238 |
| Bacteria.Tenericutes.Mollicutes.Anaeroplasmatales.Anaeroplasmataceae.Anaeroplasma | NC | 3.06116E+11 | 0.0238 |
| Bacteria.Tenericutes.Mollicutes.Anaeroplasmatales | NC | 3.06098E+11 | 0.0238 |
| Bacteria.Firmicutes.Bacilli.Bacillales.Unknown | NC | 29651525207 | 0.0062 |
| Bacteria.Firmicutes.Bacilli.Bacillales.Unknown.Unknown.Unknown | NC | 2.91065E+11 | 0.0062 |
| Bacteria.Firmicutes.Bacilli.Bacillales.Unknown.Unknown | NC | 2.90578E+11 | 0.0062 |
| Bacteria.Firmicutes.Bacilli.Bacillales | NC | 2.90537E+11 | 0.0062 |
| Bacteria.Proteobacteria.Gammaproteobacteria.Pasteurellales | NC | 2.39605E+11 | 0.0495 |
| Bacteria.Proteobacteria.Gammaproteobacteria.Pasteurellales.Pasteurellaceae.Haemophilus | NC | 2.39605E+11 | 0.0495 |
| Bacteria.Proteobacteria.Gammaproteobacteria.Pasteurellales.Pasteurellaceae | NC | 2.39605E+11 | 0.0495 |
| Bacteria.Proteobacteria.Betaproteobacteria.Burkholderiales.Unknown.Unknown | NC | 2.32998E+11 | 0.0280 |
| Bacteria.Proteobacteria.Betaproteobacteria.Burkholderiales.Unknown.Unknown.Unknown | NC | 2.32813E+11 | 0.0280 |
| Bacteria.Proteobacteria.Betaproteobacteria.Burkholderiales.Unknown | NC | 2.32273E+11 | 0.0280 |
| Bacteria.Bacteroidetes.Bacteroidia.Bacteroidales.Porphyromonadaceae.Odoribacter | HC | 3.26635E+11 | 0.0417 |
| Bacteria.Bacteroidetes.Bacteroidia.Bacteroidales.Rikenellaceae.Alistipes.Multi_affiliation | HC | 3.44015E+11 | 0.0395 |
| Bacteria.Firmicutes.Clostridia.Clostridiales.Ruminococcaceae.Ruminococcus | HC | 38209419415 | 0.0490 |
| Bacteria.Bacteroidetes.Bacteroidia.Bacteroidales.Bacteroidaceae.Bacteroides.Multi_affiliation | HC | 4.23138E+11 | 0.0251 |

**Table S2. Faecal bacterial characterization at genus level in HC and NC subjects.** Mean and standard deviation of identified genus relative abundance are shown. Original p-values and q-values obtained after p-adjustment for multiple testing are shown. Those genera with a p-value ≤ 0.05 are in bold.

| **Phylum** | **Class** | **Order** | **Family** | **Genus** | **HC** | **NC** | **p-value** | **q-value** |
| --- | --- | --- | --- | --- | --- | --- | --- | --- |
| *Actinobacteria* | *Actinobacteria* | *Actinomycetales* | *Actinomycetaceae* | *Actinomyces* | 0.0017 ± 0.0027 | 0.0014 ± 0.0031 | 0.655 | 0.9724 |
|  |  |  | *Micrococcaceae* | *Rothia* | 0.0144 ± 0.0605 | 0 ± 0 | 0.296 | 0.8726 |
|  |  | *Bifidobacteriales* | *Bifidobacteriaceae* | *Bifidobacterium* | 0.8971 ± 1.1361 | 0.9852 ± 0.886 | 0.563 | 0.954 |
|  |  | *Coriobacteriales* | *Coriobacteriaceae* | *Collinsella* | 0.2659 ± 0.4825 | 0.1783 ± 0.121 | 0.821 | 0.9892 |
|  |  |  |  | *Eggerthella* | 0.0363 ± 0.0548 | 0.0112 ± 0.0162 | 1.000 | 1 |
|  |  |  |  | *Enterorhabdus* | 0.0264 ± 0.0432 | 0.0061 ± 0.0088 | 0.439 | 0.8726 |
|  |  |  |  | *Slackia* | 0.0243 ± 0.0764 | 0.033 ± 0.0358 | 0.100 | 0.8726 |
| *Bacteroidetes* | *Bacteroidia* | *Bacteroidales* | *Bacteroidaceae* | *Bacteroides* | 23.9979 ± 16.3864 | 17.9768 ± 16.3553 | 0.364 | 0.8726 |
|  |  | *Bacteroidales* | *Porphyromonadaceae* | *Barnesiella* | 2.1842 ± 2.9046 | 3.1929 ± 3.3255 | 0.397 | 0.8726 |
|  |  |  |  | *Butyricimonas* | 0.2952 ± 0.2841 | 0.1678 ± 0.1256 | 0.349 | 0.8726 |
|  |  |  |  | ***Odoribacter*** | **0.5103 ± 0.7827** | **0.1584 ± 0.1138** | **0.044** | **0.8573** |
|  |  |  |  | *Parabacteroides* | 1.426 ± 1.1395 | 0.7432 ± 0.6286 | 0.150 | 0.8726 |
|  |  | *Bacteroidales* | *Prevotellaceae* | *Alloprevotella* | 0.4806 ± 1.5426 | 0.5055 ± 0.9959 | 0.702 | 0.9892 |
|  |  |  |  | *Hallella* | 0.0248 ± 0.0679 | 0.1338 ± 0.1671 | 0.186 | 0.8726 |
|  |  |  |  | *Paraprevotella* | 0.2784 ± 0.3967 | 0.6726 ± 0.7494 | 0.113 | 0.8726 |
|  |  |  |  | *Prevotella* | 17.1022 ± 20.7983 | 21.4225 ± 25.9141 | 0.894 | 1 |
|  |  | *Bacteroidales* | *Rikenellaceae* | *Alistipes* | 2.2731 ± 2.4952 | 1.0155 ± 1.1191 | 0.104 | 0.8726 |
| *Firmicutes* | *Bacilli* | *Lactobacillales* | *Carnobacteriaceae* | *Granulicatella* | 0.0012 ± 0.0028 | 0.0031 ± 0.0064 | 0.360 | 0.8726 |
|  |  |  | *Enterococcaceae* | *Enterococcus* | 0.0009 ± 0.0032 | 0 ± 0 | 0.250 | 0.8726 |
|  |  |  | *Lactobacillaceae* | *Lactobacillus* | 0.0477 ± 0.1422 | 0.0431 ± 0.1294 | 0.295 | 0.8726 |
|  |  |  | *Streptococcaceae* | *Streptococcus* | 0.164 ± 0.3137 | 0.3982 ± 1.0248 | 0.751 | 0.9892 |
|  | *Clostridia* | *Clostridiales* | *Clostridiaceae 1* | *Clostridium sensu stricto* | 0.0006 ± 0.0021 | 0 ± 0 | 0.568 | 0.954 |
|  |  | *Clostridiales* | *Lachnospiraceae* | *Anaerostipes* | 0.0111 ± 0.0137 | 0.018 ± 0.0187 | 0.435 | 0.8726 |
|  |  |  |  | *Blautia* | 1.8644 ± 1.281 | 1.7801 ± 0.945 | 0.861 | 0.9892 |
|  |  |  |  | *Clostridium XlVa* | 0.9759 ± 1.0406 | 0.8174 ± 0.5978 | 0.786 | 0.9892 |
|  |  |  |  | *Clostridium XlVb* | 0.0713 ± 0.0617 | 0.0713 ± 0.075 | 0.186 | 0.8726 |
|  |  |  |  | *Coprococcus* | 0.7404 ± 0.5507 | 0.5545 ± 0.6078 | 0.209 | 0.8726 |
|  |  |  |  | *Dorea* | 0.8044 ± 0.7367 | 0.756 ± 0.4492 | 0.756 | 0.9892 |
|  |  |  |  | *Howardella* | 0.0135 ± 0.019 | 0.0115 ± 0.0191 | 0.962 | 1 |
|  |  |  |  | *Roseburia* | 2.8373 ± 2.962 | 1.4068 ± 1.1268 | 0.283 | 0.8726 |
|  |  |  |  | *Ruminococcus2* | 0.3707 ± 0.2795 | 0.3501 ± 0.2011 | 0.843 | 0.9892 |
|  |  | *Clostridiales* | *Peptostreptococcaceae* | *Clostridium XI* | 0.0891 ± 0.1865 | 0.0555 ± 0.065 | 0.965 | 1 |
|  |  | *Clostridiales* | *Ruminococcaceae* | *Acetivibrio* | 0.0293 ± 0.0715 | 0.0702 ± 0.1979 | 0.823 | 0.9892 |
|  |  |  |  | *Anaerotruncus* | 0.0211 ± 0.0221 | 0.0069 ± 0.0089 | 0.056 | 0.8573 |
|  |  |  |  | *Butyricicoccus* | 0.2158 ± 0.2227 | 0.1143 ± 0.0731 | 0.372 | 0.8726 |
|  |  |  |  | *Clostridium IV* | 0.5959 ± 0.707 | 0.7363 ± 0.8458 | 0.859 | 0.9892 |
|  |  |  |  | *Faecalibacterium* | 8.6524 ± 4.0486 | 9.5987 ± 6.1103 | 0.620 | 0.954 |
|  |  |  |  | *Flavonifractor* | 0.0512 ± 0.0457 | 0.0497 ± 0.0382 | 0.929 | 1 |
|  |  |  |  | *Gemmiger* | 0.5498 ± 0.6099 | 0.3416 ± 0.4227 | 0.397 | 0.8726 |
|  |  |  |  | *Oscillibacter* | 2.9934 ± 4.6872 | 1.7635 ± 1.8335 | 0.657 | 0.9724 |
|  |  |  |  | *Pseudoflavonifractor* | 0.0367 ± 0.0386 | 0.0462 ± 0.0622 | 0.928 | 1 |
|  |  |  |  | ***Ruminococcus*** | **2.3251 ± 2.0954** | **1.1199 ± 1.5597** | **0.049** | **0.8573** |
|  |  |  |  | *Saccharofermentans* | 0.0226 ± 0.0924 | 0.0134 ± 0.0401 | 0.676 | 0.9818 |
|  |  |  |  | *Sporobacter* | 0.016 ± 0.0246 | 0.0288 ± 0.0396 | 0.395 | 0.8726 |
|  | *Erysipelotrichia* | *Erysipelotrichales* | *Erysipelotrichaceae* | *Turicibacter* | 0.0225 ± 0.0665 | 0.0085 ± 0.0118 | 0.453 | 0.8726 |
|  | *Negativicutes* | *Selenomonadales* | *Acidaminococcaceae* | *Acidaminococcus* | 0.2631 ± 0.6834 | 0.4434 ± 1.3216 | 0.834 | 0.9892 |
|  |  |  |  | *Phascolarctobacterium* | 3.74 ± 3.6694 | 7.0468 ± 8.2741 | 0.476 | 0.8729 |
|  |  | *Selenomonadales* | *Veillonellaceae* | *Allisonella* | 0.0085 ± 0.0137 | 0.0132 ± 0.0132 | 0.238 | 0.8726 |
|  |  |  |  | *Dialister* | 0.681 ± 0.9973 | 1.1315 ± 1.8869 | 0.856 | 0.9892 |
|  |  |  |  | *Megamonas* | 0.3277 ± 1.4955 | 0 ± 0 | 0.328 | 0.8726 |
|  |  |  |  | *Megasphaera* | 0.2945 ± 1.3227 | 2.1116 ± 6.0364 | 0.977 | 1 |
|  |  |  |  | *Mitsuokella* | 0.2254 ± 0.7186 | 0.1988 ± 0.3515 | 0.592 | 0.954 |
|  |  |  |  | *Multi-affiliation* | 10.0635 ± 5.4868 | 8.5511 ± 4.0611 | 0.465 | 0.8726 |
|  |  |  |  | *Veillonella* | 0.1931 ± 0.4416 | 0.1573 ± 0.2167 | 0.856 | 0.9892 |
| *Lentisphaerae* | *Lentisphaeria* | *Victivallales* | *Victivallaceae* | *Victivallis* | 0.0087 ± 0.0182 | 0.0305 ± 0.0556 | 0.459 | 0.8726 |
| *Proteobacteria* | *Alphaproteobacteria* | *Caulobacterales* | *Caulobacteraceae* | *Phenylobacterium* | 0 ± 0 | 0.0003 ± 0.001 | 0.347 | 0.8726 |
|  |  | *Rhizobiales* | *Methylobacteriaceae* | *Methylobacterium* | 0.0017 ± 0.007 | 0 ± 0 | 0.282 | 0.8726 |
|  |  | *Rhodobacterales* | *Rhodobacteraceae* | *Paracoccus* | 0.0009 ± 0.0042 | 0 ± 0 | 0.329 | 0.8726 |
|  |  | *Sphingomonadales* | *Sphingomonadaceae* | *Sphingomonas* | 0.0002 ± 0.0007 | 0.0025 ± 0.0054 | 0.140 | 0.8726 |
|  | *Betaproteobacteria* | *Burkholderiales* | *Comamonadaceae* | *Pelomonas* | 0.0005 ± 0.0015 | 0.0004 ± 0.0013 | 0.931 | 1 |
|  |  |  | *Oxalobacteraceae* | *Oxalobacter* | 0.0086 ± 0.0176 | 0.0181 ± 0.0224 | 0.373 | 0.8726 |
|  |  |  | *Sutterellaceae* | *Parasutterella* | 0.3132 ± 0.4398 | 0.2138 ± 0.4745 | 0.365 | 0.8726 |
|  |  |  |  | *Sutterella* | 0.583 ± 1.0673 | 0.3743 ± 0.6803 | 0.612 | 0.954 |
|  | *Deltaproteobacteria* | *Desulfovibrionales* | *Desulfovibrionaceae* | *Bilophila* | 0.118 ± 0.0953 | 0.0723 ± 0.0855 | 0.124 | 0.8726 |
|  |  |  |  | *Desulfovibrio* | 0.0189 ± 0.0567 | 0.1027 ± 0.1684 | 0.383 | 0.8726 |
|  | *Epsilonproteobac* | *Campylobacterales* | *Helicobacteraceae* | *Helicobacter* | 0.0001 ± 0.0007 | 0 ± 0 | 0.329 | 0.8726 |
|  | *Gammaproteobacteria* | *Aeromonadales* | *Succinivibrionaceae* | *Succinivibrio* | 1.7649 ± 8.0731 | 0.0142 ± 0.029 | 0.577 | 0.954 |
|  |  | *Enterobacteriales* | *Enterobacteriaceae* | *Enterobacter* | 0.0968 ± 0.3807 | 0.0049 ± 0.0148 | 1.000 | 1 |
|  |  |  |  | *Escherichia/Shigella* | 0.1577 ± 0.4522 | 0.0195 ± 0.0238 | 0.854 | 0.9892 |
|  |  | *Pasteurellales* | *Pasteurellaceae* | ***Haemophilus*** | **0.0416 ± 0.1385** | **0.0779 ± 0.1656** | **0.049** | **0.8573** |
|  |  | *Pseudomonadales* | *Moraxellaceae* | *Acinetobacter* | 0.0002 ± 0.0008 | 0 ± 0 | 0.329 | 0.8726 |
|  |  |  |  | *Enhydrobacter* | 0.0003 ± 0.0014 | 0.0014 ± 0.0041 | 0.530 | 0.9494 |
|  |  |  | *Pseudomonadaceae* | *Pseudomonas* | 0.3714 ± 1.6513 | 0.0052 ± 0.0116 | 0.609 | 0.954 |
| *Synergistetes* | *Synergistia* | *Synergistales* | *Synergistaceae* | *Cloacibacillus* | 0.0264 ± 0.1172 | 0.001 ± 0.0029 | 1.000 | 1 |
| *Tenericutes* | *Mollicutes* | *Anaeroplasmatales* | *Anaeroplasmataceae* | ***Anaeroplasma*** | **0.0021 ± 0.006** | **0.2198 ± 0.4584** | **0.026** | **0.8573** |
| *Unknown* | *Unknown* | *Unknown* | *Unknown* | *Unknown* | 7.3049 ± 4.3483 | 11.8188 ± 14.2388 | 0.756 | 0.9892 |
| *Verrucomicrobia* | *Verrucomicrobiae* | *Verrucomicrobiales* | *Verrucomicrobiaceae* | *Akkermansia* | 0.0203 ± 0.0513 | 0.001 ± 0.0021 | 0.435 | 0.8726 |

**Table S3. Daily nutrients and energy intake in HC and NC subjects.** Mean and standard deviation of each variable are shown. Variables of each group were compared by unpaired t-test and corrected for multiple comparisons. p-value and q-value of each comparison are shown. Abbreviations: saturated fatty acids (SFA), monounsaturated fatty acids (MFA) and polyunsaturated fatty acids (PUFA).

| **Dietary paramater** | | **HC** | **NC** | **p-value** | **q-value** |
| --- | --- | --- | --- | --- | --- |
| **Kcal** |  | 1812 ± 471.9 | 2054.4 ± 126.7 | 0.038 | 0.408 |
| **Protein (g)** |  | 80 ± 32.9 | 83.4 ± 21.6 | 0.742 | 0.835 |
| **Carbohydrates (g)** |  | 177.7 ± 55.3 | 191.1 ± 53.5 | 0.541 | 0.717 |
|  | **Sugars (g)** | 71.4 ± 39 | 85.2 ± 40.7 | 0.403 | 0.62 |
|  | **Starch (g)** | 100.2 ± 39.7 | 97.8 ± 30.8 | 0.86 | 0.893 |
| **Fat (g)** |  | 76.1 ± 29.8 | 92.4 ± 18.6 | 0.081 | 0.408 |
|  | **SFA (g)** | 23.8 ± 13.3 | 32.3 ± 8.7 | 0.049 | 0.408 |
|  | **MFA (g)** | 31.2 ± 12.5 | 38.4 ± 9.3 | 0.097 | 0.408 |
|  | **PUFA (g)** | 10.7 ± 5.7 | 13.9 ± 4.5 | 0.121 | 0.408 |
| **Fiber (g)** |  | 17.8 ± 5.6 | 23.7 ± 6.3 | 0.057 | 0.408 |
|  | **Soluble fiber (g)** | 4 ± 2.3 | 5.3 ± 2.3 | 0.181 | 0.466 |
|  | **Insoluble fiber (g)** | 7 ± 3.4 | 9.6 ± 4 | 0.112 | 0.408 |
|  | **Soluble fiber (%)** | 36.2 ± 7.5 | 34.8 ± 5.8 | 0.584 | 0.717 |
|  | **Insoluble fiber (%)** | 63.8 ± 7.5 | 65.2 ± 5.8 | 0.584 | 0.717 |
| **Protein (% kcal)** |  | 17.1 ± 4.6 | 16.3 ± 4.3 | 0.633 | 0.743 |
| **Carbohydrates (% kcal)** |  | 40.2 ± 11.4 | 37 ± 9.5 | 0.436 | 0.62 |
|  | **Sugars (% kcal)** | 15.6 ± 7.4 | 16.4 ± 6.9 | 0.779 | 0.841 |
|  | **Starch (% kcal)** | 23.3 ± 10.6 | 19 ± 5.9 | 0.176 | 0.466 |
| **Fat (% kcal)** |  | 36.9 ± 8.2 | 40.4 ± 7.9 | 0.281 | 0.584 |
|  | **SFA (% kcal)** | 11.3 ± 4.6 | 14.2 ± 3.7 | 0.091 | 0.408 |
|  | **MFA (% kcal)** | 15.5 ± 4.2 | 16.8 ± 4.1 | 0.42 | 0.62 |
|  | **PUFA (% kcal)** | 5.3 ± 2.6 | 6.1 ± 1.9 | 0.4 | 0.62 |

**Table S4: Raw p-value and q-value after multiple testing correction for each spearman correlation between lipids biomarkers and discriminant genera in HC and NC. Q-values < 0.15 are in bold.**

|  | ***Anaeroplasma*** | | ***Haemophilus*** | | ***Odoribacter*** | | ***Ruminococcus*** | |
| --- | --- | --- | --- | --- | --- | --- | --- | --- |
|  | **p-value** | **q-value** | **p-value** | **q-value** | **p-value** | **q-value** | **p-value** | **q-value** |
| **VLDL.C** | 0.118 | 0.158 | 0.035 | **0.138** | 0.080 | 0.158 | 0.855 | 0.855 |
| **IDL.C** | 0.018 | **0.069** | 0.040 | **0.069** | 0.052 | **0.069** | 0.359 | 0.359 |
| **LDL.C** | 0.235 | 0.246 | 0.054 | 0.214 | 0.246 | 0.246 | 0.178 | 0.246 |
| **HDL.C** | 0.939 | 0.939 | 0.163 | 0.650 | 0.358 | 0.717 | 0.873 | 0.939 |
| **Total.C** | 0.109 | 0.154 | 0.034 | **0.136** | 0.115 | 0.154 | 0.434 | 0.434 |
| **VLDL.TG** | 0.103 | **0.137** | 0.033 | **0.131** | 0.071 | **0.137** | 0.893 | 0.893 |
| **IDL.TG** | 0.022 | **0.066** | 0.039 | **0.066** | 0.049 | **0.066** | 0.989 | 0.989 |
| **LDL.TG** | 0.055 | **0.103** | 0.024 | **0.098** | 0.077 | **0.103** | 0.350 | 0.350 |
| **HDL.TG** | 0.027 | **0.066** | 0.033 | **0.066** | 0.499 | 0.499 | 0.131 | 0.175 |
| **Total.TG** | 0.052 | **0.088** | 0.023 | **0.088** | 0.066 | **0.088** | 0.814 | 0.814 |
| **Non.HDL.P** | 0.071 | **0.132** | 0.007 | **0.028** | 0.099 | **0.132** | 0.414 | 0.414 |
| **Total.P/HDL.P** | 0.318 | 0.318 | 0.034 | **0.136** | 0.100 | 0.193 | 0.145 | 0.193 |
| **LDL.P/HDL.P** | 0.309 | 0.309 | 0.077 | **0.146** | 0.110 | **0.146** | 0.062 | **0.146** |
| **Total.C/HDL.C** | 0.060 | **0.080** | 0.007 | **0.026** | 0.017 | **0.035** | 0.406 | 0.406 |
| **APOA1** | 0.417 | 0.833 | 0.803 | 0.957 | 0.185 | 0.738 | 0.957 | 0.957 |
| **VLDL.P** | 0.109 | **0.146** | 0.043 | **0.137** | 0.068 | **0.137** | 0.877 | 0.877 |
| **Large.VLDL.P** | 0.135 | 0.180 | 0.034 | **0.120** | 0.060 | **0.120** | 0.899 | 0.899 |
| **Medium.VLDL.P** | 0.080 | **0.107** | 0.027 | **0.107** | 0.070 | **0.107** | 0.959 | 0.959 |
| **Small.VLDL.P** | 0.125 | 0.167 | 0.050 | **0.124** | 0.062 | **0.124** | 0.792 | 0.792 |
| **LDL.P** | 0.127 | 0.177 | 0.016 | **0.065** | 0.133 | 0.177 | 0.296 | 0.296 |
| **Large.LDL.P** | 0.243 | 0.505 | 0.505 | 0.505 | 0.496 | 0.505 | 0.256 | 0.505 |
| **Medium.LDL.P** | 0.154 | 0.154 | 0.029 | **0.115** | 0.147 | 0.154 | 0.126 | 0.154 |
| **Small.LDL.P** | 0.082 | 0.165 | 0.007 | **0.028** | 0.151 | 0.201 | 0.645 | 0.645 |
| **HDL.P** | 0.125 | 0.501 | 0.764 | 0.933 | 0.843 | 0.933 | 0.933 | 0.933 |
| **Large.HDL.P** | 0.513 | 0.513 | 0.314 | 0.513 | 0.119 | 0.476 | 0.413 | 0.513 |
| **Medium.HDL.P** | 0.681 | 0.908 | 0.336 | 0.672 | 0.142 | 0.567 | 0.966 | 0.966 |
| **Small.HDL.P** | 0.100 | 0.306 | 0.222 | 0.306 | 0.230 | 0.306 | 0.901 | 0.901 |
| **VLDL.Z** | 0.955 | 0.955 | 0.406 | 0.802 | 0.602 | 0.802 | 0.240 | 0.802 |
| **LDL.Z** | 0.761 | 0.761 | 0.036 | **0.145** | 0.526 | 0.761 | 0.643 | 0.761 |
| **HDL.Z** | 0.097 | **0.138** | 0.071 | **0.138** | 0.103 | **0.138** | 0.923 | 0.923 |

**Table S5. Co-ocurrence correlation of genera differently represented in HC and NC subjects.** Spearman’s rho, original p-values and q-values after correction for multiple analysis are indicated for each correlation. Only correlations with q-value < 0.15 are shown.

|  | **Bacteria co-ocurrence** | **Spearman's *rho*** | **p-value** | **q-value** |
| --- | --- | --- | --- | --- |
| ***Anaeroplasma*** | *Acetivibrio* | 0,532 | 0,002 | 0.012 |
|  | *Acidaminococcus* | 0,427 | 0,019 | 0.131 |
|  | *Clostridium IV* | 0,495 | 0,005 | 0.025 |
|  | *Desulfovibrio* | 0,374 | 0,042 | 0.145 |
|  | *Granulicatella* | 0,468 | 0,009 | 0.063 |
|  | *Oxalobacter* | 0,387 | 0,035 | 0.120 |
|  | *Roseburia* | -0,403 | 0,027 | 0.095 |
| ***Haemophilus*** | *Actinomyces* | 0,415 | 0,023 | 0.058 |
|  | *Clostridium XI* | 0,473 | 0,008 | 0.025 |
|  | *Desulfovibrio* | 0,440 | 0,015 | 0.059 |
|  | *Slackia* | 0,392 | 0,032 | 0.128 |
|  | *Veillonella* | 0,591 | 0,001 | 0.035 |
| ***Odoribacter*** | *Alistipes* | 0,643 | 0,000 | 0.000 |
|  | *Butyricimonas* | 0,546 | 0,002 | 0.005 |
|  | *Oscillibacter* | 0,443 | 0,014 | 0.019 |
|  | *Parabacteroides* | 0,478 | 0,008 | 0.128 |
|  | *Ruminococcus* | 0,459 | 0,011 | 0.02 |
|  | *Turicibacter* | -0,469 | 0,009 | 0.049 |
| ***Ruminococcus*** | *Acetivibrio* | 0,422 | 0,020 | 0.065 |
|  | *Alistipes* | 0,572 | 0,001 | 0.004 |
|  | *Butyricimonas* | 0,400 | 0,029 | 0.076 |
|  | *Gemmiger* | 0,380 | 0,038 | 0.123 |
|  | *Granulicatella* | 0,372 | 0,043 | 0.114 |
|  | *Odoribacter* | 0,459 | 0,011 | 0.027 |
|  | *Oscillibacter* | 0,538 | 0,002 | 0.008 |
|  | *Pseudoflavonifractor* | 0,403 | 0,027 | 0.071 |
|  | *Rothia* | 0,437 | 0,016 | 0.101 |
|  | *Succinivibrio* | - 0,464 | 0,010 | 0.064 |

**Table S6: Raw p-value and q-value after multiple testing correction for each spearman correlation between lipids biomarkers and faecal metabolites in HC and NC. Q-values < 0.15 are in bold.**

|  | **Succinic.Ac** | | **Totals.SCFAs** | | **Acetic.Ac** | | **Propionic.Ac** | | **Butyric.Ac** | | **Isobutyric.Ac** | | **Isovaleric.Ac** | |
| --- | --- | --- | --- | --- | --- | --- | --- | --- | --- | --- | --- | --- | --- | --- |
|  | **p-value** | **q-value** | **p-value** | **q-value** | **p-value** | **q-value** | **p-value** | **q-value** | **p-value** | **q-value** | **p-value** | **q-value** | **p-value** | **q-value** |
| **VLDL.C** | 0.783 | 0.942 | 0.877 | 0.942 | 0.71 | 0.942 | 0.334 | 0.942 | 0.791 | 0.942 | 0.082 | 0.409 | 0.847 | 0.864 |
| **IDL.C** | 0.326 | 0.449 | 0.405 | 0.449 | 0.025 | **0.15** | 0.043 | **0.15** | 0.278 | 0.449 | 0.185 | 0.324 | 0.359 | 0.418 |
| **LDL.C** | 0.875 | 0.992 | 0.33 | 0.77 | 0.299 | 0.77 | 0.091 | 0.638 | 0.535 | 0.935 | 0.027 | **0.123** | 0.437 | 0.949 |
| **HDL.C** | 0.914 | 0.974 | 0.939 | 0.974 | 0.974 | 0.974 | 0.621 | 0.974 | 0.415 | 0.974 | 0.522 | 0.832 | 0.644 | 0.901 |
| **Total.C** | 0.691 | 0.962 | 0.362 | 0.894 | 0.511 | 0.894 | 0.079 | 0.552 | 0.451 | 0.894 | 0.095 | 0.34 | 0.521 | 0.948 |
| **VLDL.TG** | 0.76 | 0.973 | 0.939 | 0.973 | 0.708 | 0.973 | 0.257 | 0.899 | 0.888 | 0.973 | 0.061 | 0.425 | 0.88 | 0.88 |
| **IDL.TG** | 0.654 | 0.663 | 0.655 | 0.663 | 0.195 | 0.456 | 0.137 | 0.456 | 0.663 | 0.663 | 0.161 | 0.462 | 0.912 | 0.912 |
| **LDL.TG** | 0.639 | 0.815 | 0.417 | 0.815 | 0.215 | 0.753 | 0.055 | 0.384 | 0.657 | 0.815 | 0.034 | 0.179 | 0.291 | 0.859 |
| **HDL.TG** | 0.175 | 0.613 | 0.17 | 0.613 | 0.901 | 0.901 | 0.296 | 0.691 | 0.593 | 0.831 | 0.877 | 0.894 | 0.88 | 1 |
| **Total.TG** | 0.738 | 0.952 | 0.963 | 0.963 | 0.642 | 0.952 | 0.292 | 0.952 | 0.816 | 0.952 | 0.139 | 0.575 | 0.843 | 0.982 |
| **NonHDLP** | 0.51 | 0.83 | 0.244 | 0.83 | 0.531 | 0.83 | 0.155 | 0.83 | 0.775 | 0.89 | 0.066 | 0.465 | 0.194 | 0.678 |
| **TotalP/HDLP** | 0.73 | 0.73 | 0.279 | 0.651 | 0.268 | 0.651 | 0.708 | 0.73 | 0.38 | 0.665 | 0.037 | 0.256 | 0.121 | 0.281 |
| **LDLP/HDLP** | 0.851 | 0.851 | 0.355 | 0.79 | 0.229 | 0.79 | 0.665 | 0.851 | 0.451 | 0.79 | 0.016 | **0.115** | 0.27 | 0.629 |
| **TotalC/HDLC** | 0.656 | 0.656 | 0.629 | 0.656 | 0.458 | 0.656 | 0.448 | 0.656 | 0.607 | 0.656 | 0.014 | **0.099** | 0.067 | 0.233 |
| **APOA1** | 0.905 | 0.905 | 0.899 | 0.905 | 0.587 | 0.905 | 0.611 | 0.905 | 0.784 | 0.905 | 0.302 | 0.86 | 0.27 | 0.472 |
| **VLDL.P** | 0.791 | 0.986 | 0.986 | 0.986 | 0.675 | 0.986 | 0.269 | 0.942 | 0.844 | 0.986 | 0.083 | 0.494 | 0.948 | 0.948 |
| **Large.VLDLP** | 0.855 | 0.897 | 0.897 | 0.897 | 0.714 | 0.897 | 0.367 | 0.897 | 0.857 | 0.897 | 0.039 | 0.272 | 0.776 | 0.843 |
| **Medium.VLDLP** | 0.702 | 0.988 | 0.857 | 0.988 | 0.849 | 0.988 | 0.303 | 0.988 | 0.988 | 0.988 | 0.041 | 0.284 | 0.708 | 0.826 |
| **Small.VLDLP** | 0.866 | 0.951 | 0.919 | 0.951 | 0.704 | 0.951 | 0.291 | 0.951 | 0.851 | 0.951 | 0.088 | 0.574 | 0.948 | 0.948 |
| **LDLP** | 0.685 | 0.813 | 0.257 | 0.813 | 0.37 | 0.813 | 0.1 | 0.7 | 0.58 | 0.813 | 0.032 | **0.12** | 0.359 | 0.837 |
| **Large.LDLP** | 0.966 | 0.966 | 0.76 | 0.966 | 0.574 | 0.966 | 0.173 | 0.884 | 0.319 | 0.884 | 0.195 | 0.448 | 0.313 | 0.729 |
| **Medium.LDLP** | 0.864 | 0.864 | 0.535 | 0.864 | 0.297 | 0.864 | 0.145 | 0.864 | 0.667 | 0.864 | 0.018 | **0.123** | 0.613 | 1 |
| **Small.LDLP** | 0.494 | 0.691 | 0.178 | 0.647 | 0.37 | 0.647 | 0.279 | 0.647 | 0.771 | 0.899 | 0.119 | 0.627 | 0.291 | 0.843 |
| **HDLP** | 0.519 | 0.845 | 0.724 | 0.845 | 0.708 | 0.845 | 0.337 | 0.787 | 0.279 | 0.787 | 0.951 | 0.951 | 0.912 | 0.982 |
| **Large.HDLP** | 0.164 | 0.399 | 0.186 | 0.399 | 0.228 | 0.399 | 0.016 | **0.113** | 0.651 | 0.651 | 0.033 | 0.223 | 0.552 | 0.678 |
| **Medium.HDLP** | 0.37 | 0.912 | 0.391 | 0.912 | 0.873 | 0.95 | 0.95 | 0.95 | 0.673 | 0.95 | 0.095 | 0.487 | 0.23 | 0.322 |
| **Small.HDLP** | 0.595 | 0.833 | 0.529 | 0.833 | 0.952 | 0.969 | 0.092 | 0.646 | 0.318 | 0.833 | 0.522 | 0.642 | 0.912 | 0.982 |
| **VLDLZ** | 0.538 | 0.908 | 0.908 | 0.908 | 0.507 | 0.908 | 0.448 | 0.908 | 0.514 | 0.908 | 0.527 | 0.857 | 0.133 | 0.826 |
| **LDLZ** | 0.188 | 0.639 | 0.421 | 0.639 | 0.718 | 0.718 | 0.491 | 0.639 | 0.548 | 0.639 | 0.335 | 0.781 | 0.678 | 0.678 |
| **HDLZ** | 0.817 | 0.817 | 0.341 | 0.752 | 0.644 | 0.752 | 0.514 | 0.752 | 0.644 | 0.752 | 0.011 | **0.077** | 0.463 | 0.81 |

**Figure S1. Correlation between dietary features and differential genus in HC and NC subjects.** Correlations were assessed considering data from both groups together. Correlations with q-values below 0.15 after adjustment for multiple analysis are highlighted with an ellipse. The colour and slope of the ellipse indicate magnitude of correlation, with Spearman’s rho value superimposed on the ellipse. The ellipses of positive correlations are shown in blue and the negative correlations in red. Correlations with q-value > 0.15 are in white. Abbreviations: saturated fatty acids (SFA), monounsaturated fatty acids (MFA) and polyunsaturated fatty acids (PUFA).

**
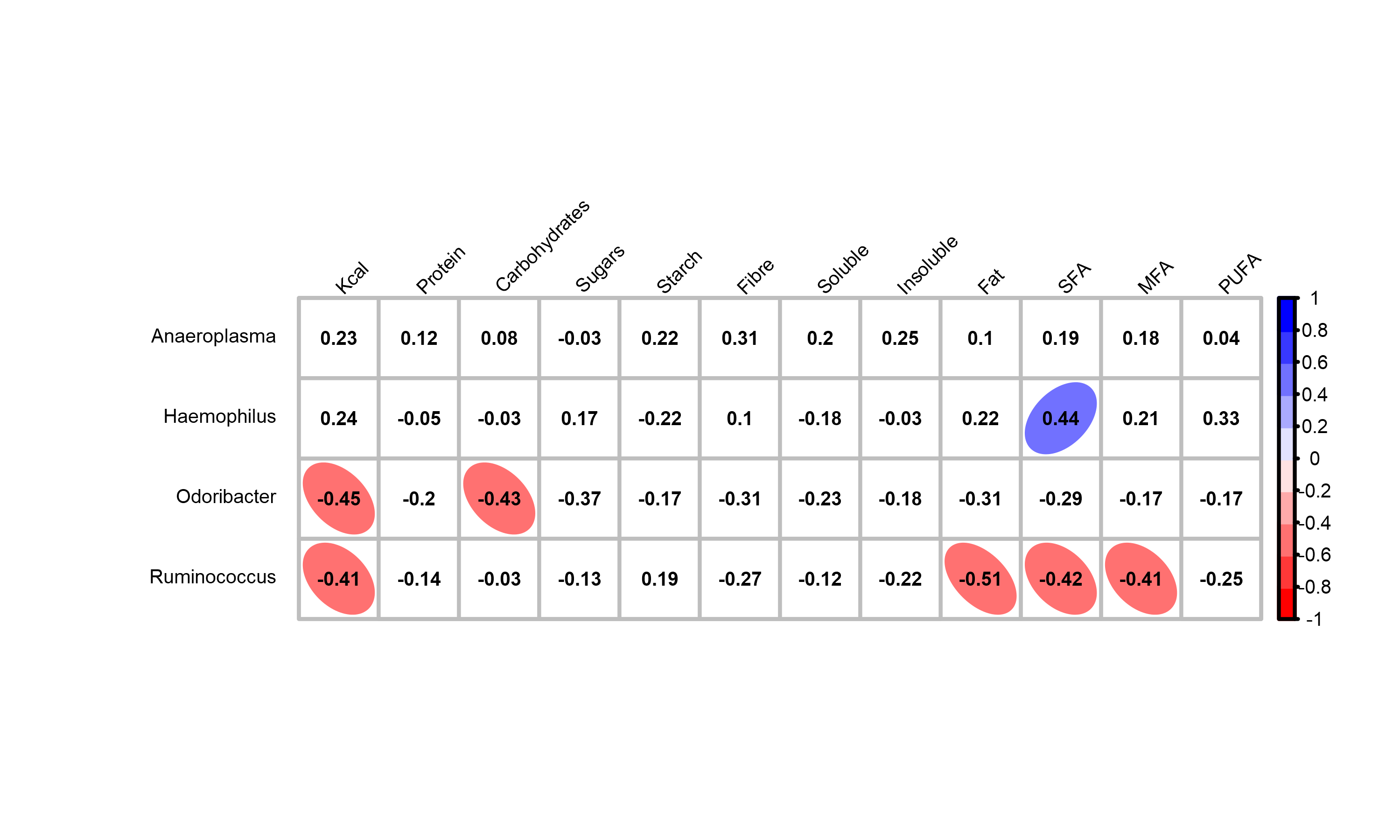
**

**Figure S2. Correlation between subject’s age and *Ruminococcus* relative abundance.** Plot of spearman’s correlation analysis taking data from hypercholesterolemic and normocholesterolemic subjects into account. Spearman’s coefficient (*rho*) and p-value are indicated on the top right of the graph.

**Figure S3. Correlation between serum lipid biomarkers and faecal bacteria at genus level.** Correlations were assessed considering data from both groups together. The colour and slope of the ellipse indicate magnitude of correlation, with Spearman’s *rho* value superimposed on the ellipse. Correlations with q-values below 0.25 after adjustment for multiple analysis are highlighted with an ellipse. The colour and slope of the ellipse indicate magnitude of correlation, with Spearman’s rho value superimposed on the ellipse. The ellipses of positive correlations are shown in blue and the negative correlations in red. Correlations with q-value > 0.25 are in white. The Phylum to which bacteria belong to is indicated by colour: *Actinobacteria* in pink, *Bacteroidetes* in blue, *Firmicutes* in light red, *Lentisphaerae* in orange and *Proteobacteria* in a green.

**
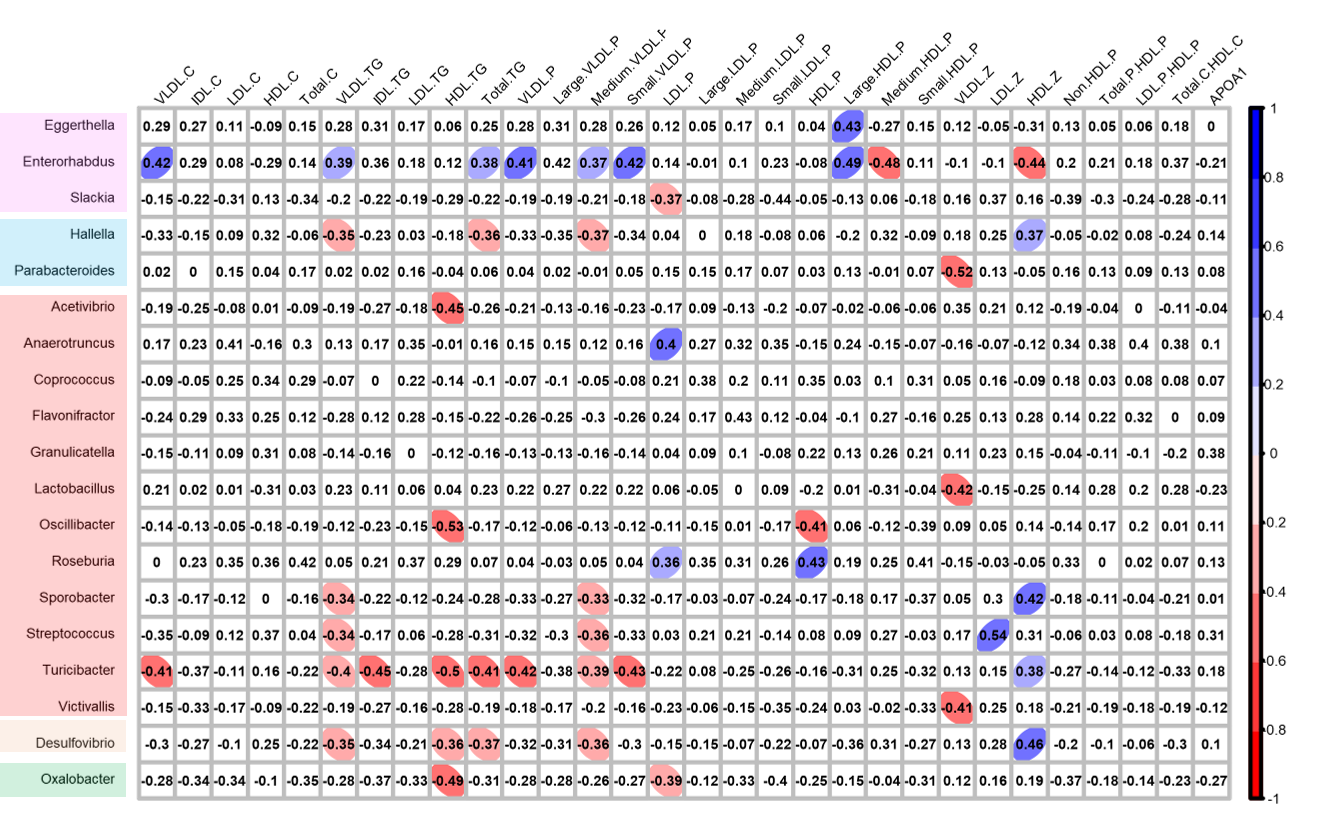
**

**Figures S4. Serum SCFAs profile of HC and NC subjects and lipid biomarkers association.** A) Total and B) individual SCFA levels observed in serum of HC and NC. P-values < 0.05 after comparisons between groups are indicated. C) Correlation between serum SCFAs abundances and lipid biomarkers levels. Correlations were assessed considering data from both groups together. Correlations with q-values below 0.15 after adjustment for multiple analysis are highlighted with an ellipse. The colour and slope of the ellipse indicate magnitude of correlation, with Spearman’s rho value superimposed on the ellipse. The ellipses of positive correlations are shown in blue and the negative correlations in red. Correlations with q-value > 0.15 are in white.


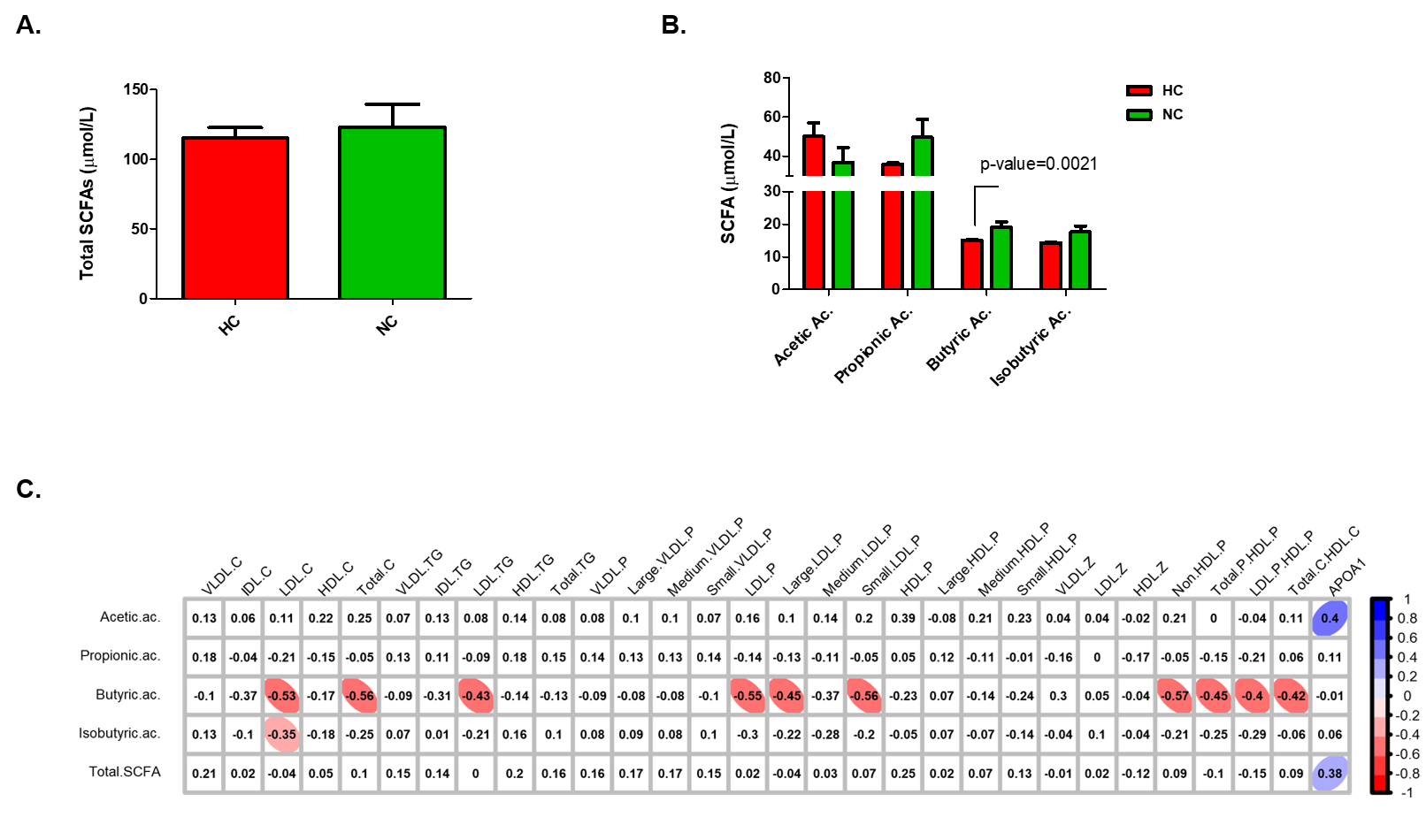


**Figures S5. Faecal profile of sterols and bile acids in HC and NC subjects and lipid biomarkers association.** A) Faecal sterols and B) bile acids profile in HC and NC. C) Correlation analysis of sterols and bile acids with the differential genera between HC and NC. Correlations were assessed considering data from both groups together. Correlations with q-values below 0.15 after adjustment for multiple analysis are highlighted with an ellipse. The colour and slope of the ellipse indicate magnitude of correlation, with Spearman’s rho value superimposed on the ellipse. The ellipses of positive correlations are shown in blue and the negative correlations in red. Correlations with q-value > 0.15 are in white.


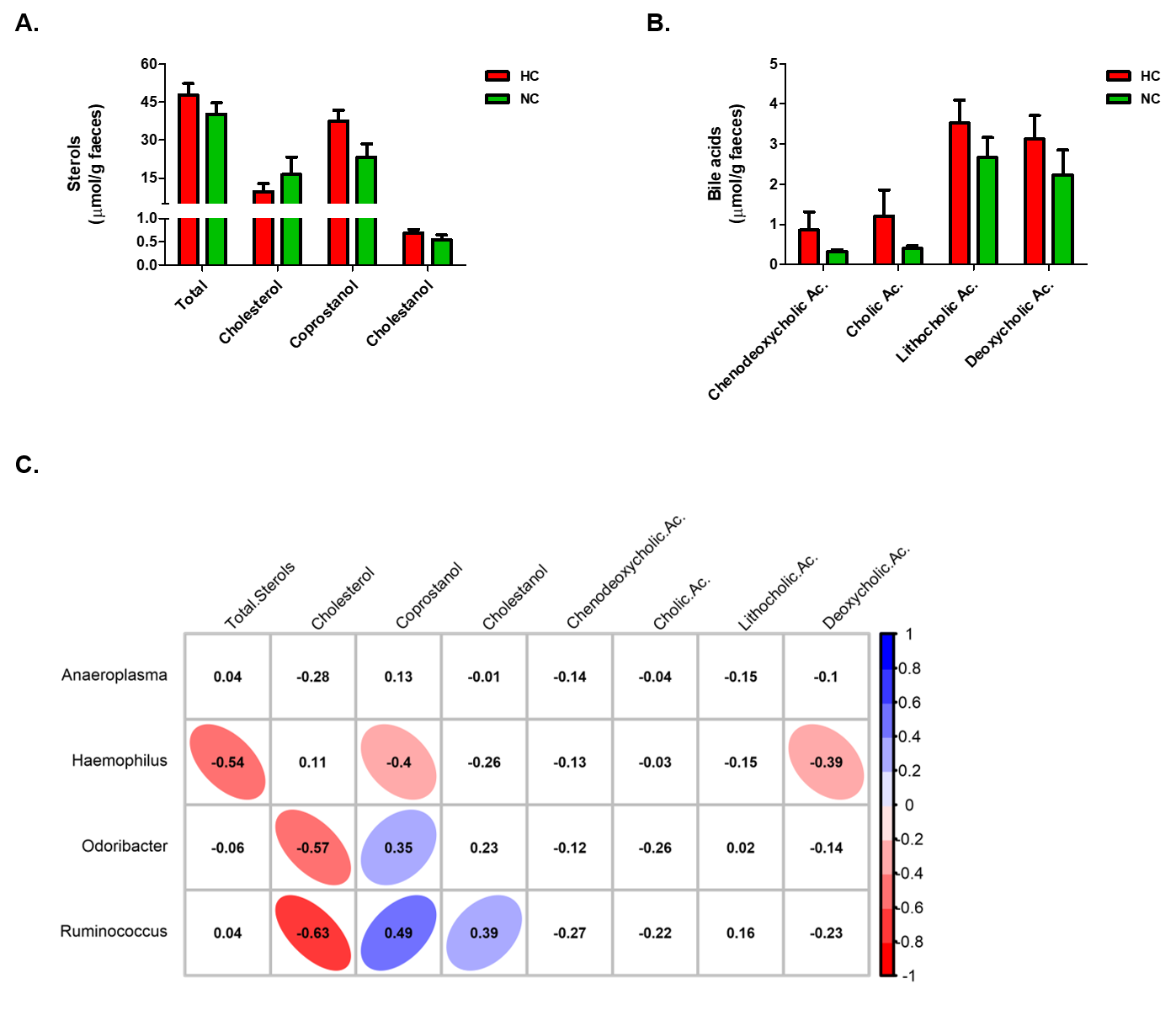

Supplement: Supplementary file 1 — Supplementary information [file 41598_2019_38874_MOESM1_ESM.docx]
